# Comparing the Cytokine Storms of COVID-19 and Pandemic Influenza

**DOI:** 10.1089/jir.2022.0029

**Published:** 2022-08-18

**Authors:** Lynette Miroslava Pacheco-Hernández, Jazmín Ariadna Ramírez-Noyola, Itzel Alejandra Gómez-García, Sergio Ignacio-Cortés, Joaquín Zúñiga, José Alberto Choreño-Parra

**Affiliations:** ^1^ Laboratory of Immunobiology and Genetics, Instituto Nacional de Enfermedades Respiratorias “Ismael Cosío Villegas,” Mexico City, Mexico.; ^2^ Tecnologico de Monterrey, Escuela de Medicina y Ciencias de la Salud, Mexico City, Mexico.; ^3^ Programa de Maestría en Ciencias de la Salud, Sección de Estudios de Posgrado e Investigación, Escuela Superior de Medicina, Instituto Politécnico Nacional, Salvador Díaz Mirón and Plan de San Luis, Mexico City, Mexico.

**Keywords:** COVID-19, SARS-CoV-2, influenza, flu, cytokine storm, cytokines

## Abstract

Emerging respiratory viruses are major health threats due to their potential to cause massive outbreaks. Over the past 2 years, the coronavirus disease 2019 (COVID-19) pandemic has caused millions of cases of severe infection and deaths worldwide. Although natural and vaccine-induced protective immune mechanisms against the severe acute respiratory syndrome coronavirus 2 (SARS-CoV-2) have been increasingly identified, the factors that determine morbimortality are less clear. Comparing the immune signatures of COVID-19 and other severe respiratory infections such as the pandemic influenza might help dissipate current controversies about the origin of their severe manifestations. As such, identifying homologies in the immunopathology of both diseases could provide targets for immunotherapy directed to block shared pathogenic mechanisms. Meanwhile, finding unique characteristics that differentiate each infection could shed light on specific immune alterations exploitable for diagnostic and individualized therapeutics for each case. In this study, we summarize immunopathological aspects of COVID-19 and pandemic influenza from the perspective of cytokine storms as the driving force underlying morbidity. Thereby, we analyze similarities and differences in the cytokine profiles of both infections, aiming to bring forward those molecules more attractive for translational medicine and drug development.

## Introduction

Outbreaks of viral pneumonia have occurred all along human history. Although the mechanism behind morbidity remained unclear for decades, current paradigms indicate that besides the microorganisms' virulence, an overdriven host immune response mediates devastating manifestations of infections. This idea has gained further notoriety after the coronavirus disease 2019 (COVID-19) pandemic. Thus, it is now accepted that the critical forms of the disease are frequently accompanied by excessive cytokine release into the circulation (hypercytokinemia) (Mehta and others 2020).

Despite advances in understanding COVID-19 pathobiology, the exact cytokine networks involved in severe manifestations and how each factor contributes to lung damage are unclear. Defining immune profiles associated with morbidity is complex due to the impact of genetic and comorbidity differences across populations. In this scenario, lessons from other respiratory infections might aid dissipating uncertainty about COVID-19 immunopathology. Influenza viruses are the prototype airborne pathogens leading to periodic epidemics of variable severity, the last occurring in 2009 after the appearance of a novel A (H1N1) subtype (Centers for Disease and Prevention 2009; Novel Swine-Origin Influenza and others 2009; Perez-Padilla and others 2009). Similar to the severe acute respiratory syndrome coronavirus 2 (SARS-CoV-2), pandemic influenza A (H1N1), hereinafter referred to as influenza, is characterized by a broad clinical spectrum encompassing critical respiratory illness with hypercytokinemia (Liu and others 2016; Thomas and others 2017).

This review compares cytokine storm syndromes (CSS) observed during COVID-19 and influenza to detect conserved immunopathogenic mechanisms underlying severe disease. Moreover, by highlighting unique immune profiles in critical COVID-19, we provide the theoretical bases for future research on specific cytokine networks implicated in pathogenesis that could be targeted through immunotherapy.

## Infectious CSS

### Mechanisms

Cytokines coordinate the immune response activation, regulation, and amplification. They have short half-life times, and their production is very regulated to prevent systemic damage (Cavaillon and others 1992). Cytokines act through common intracellular pathways to control intercellular interaction and communication, and they have autocrine, paracrine, or endocrine effects (Zhang and An 2007). Once released, cytokines induce the production of more cytokines (cytokine cascades). A cytokine storm (CS) is an increase in circulating cytokines causing acute systemic symptoms and organ dysfunction (Fajgenbaum and June 2020). The term was first used for graft-versus-host disease in 1993 (Ferrara and others 1993). Nevertheless, this phenomenon was associated with infections until the H5N1 influenza virus emergence in 2005 (Yuen and Wong 2005).

It is well known that CSS can occur in various contexts due to excessive cytokine production or inadequate anti-inflammatory responses. For instance, the hemophagocytic syndrome (HPS), also named hemophagocytic lymphohistiocytosis (HLH), is characterized by immune overstimulation. This condition can be primary and secondary according to its cause (Buyse and others 2010; Canna and Behrens 2012). Primary HLH, as in the case of familial HLH, derives from genetic mutations altering the function of natural killer (NK) cells and cytotoxic T cells (Stepp and others 1999). However, it also includes other inherited immunodeficiencies, such as the Chédiak–Higashi syndrome, Griscelli syndrome, and type II Hermansky–Pudlak syndrome (Emmenegger and others 2005). The typical cause of secondary HPS is infections, especially related to the Epstein–Barr virus, human immunodeficiency virus, herpesvirus 1, bacteria, and fungi.

Nonetheless, it can also be associated with autoimmune diseases and malignancies such as leukemia and lymphoma (Al-Samkari and Berliner 2018). Macrophage activation syndrome is also a secondary HPS associated with rheumatic diseases, especially systemic juvenile idiopathic arthritis, systemic lupus erythematosus, and adult-onset Still's disease (Fukaya and others 2008). Also, the cytokine release syndrome is a class of CSS occurring in patients with B cell malignancies after chimeric antigen receptor T cell immunotherapy (Porter and others 2015).

Typical manifestations of hypercytokinemia include fever, malaise, anorexia, hypotension, hypoxia, arthralgia/myalgia, nausea, diarrhea, tachycardia, tachypnea, altered mental status, diffuse lymphadenopathy, hepatosplenomegaly, rash, pulmonary edema, pneumonitis, and renal failure. There are also common laboratory findings characteristic of an acute-phase response such as leukocytosis/leukopenia, thrombocytosis/thrombocytopenia, anemia, increased C-reactive protein (CRP), ferritin and D-dimer levels, prolonged prothrombin time, decreased erythrocyte sedimentation rate, hypertriglyceridemia, and hypoalbuminemia (Fajgenbaum and June 2020; Lukan 2020). All these changes are driven by the biological activities of specific cytokines usually overproduced during a CSS.

### Sepsis exemplifies an infectious CSS

Sepsis illustrates the clinical consequences of hypercytokinemia during infections and is an example to understand the pathobiology of CSS (Cohen 2002; Schulte and others 2013). Indeed, influenza and COVID-19 also meet the criteria for sepsis, defined as a life-threatening organ dysfunction caused by a dysregulated host response to infection (Singer and others 2016). Clinical manifestations associated with sepsis resemble other CSS and include an increased respiratory rate, altered mental status, and hypotension. Septic shock is a subset of sepsis, in which underlying circulatory and cellular/metabolic abnormalities are that profound to increase mortality substantially. It is characterized by hypotension refractory to fluid resuscitation and increased serum lactate levels (Singer and others 2016).

Sepsis has been intensively investigated for decades, allowing immunologists to discover fundamental mechanisms of immune activation and regulation (Opal 2011). All responses against infections initiate when the innate immune system detects pathogen-associated molecular patterns (PAMPs) expressed by invading microorganisms using pattern recognition receptors (PRRs) such as the Toll-like receptors (TLRs), NOD-like receptors (NLRs), retinoic-acid-inducible gene 1 (RIG-1), among others (Eisenbarth and Flavell 2009). These receptors initiate signaling pathways that culminate in reactive oxygen species and reactive nitrogen species (ROS and RNS) production, complement activation, phagocytosis, chemotaxis, and cytokine expression, increasing blood supply and leukocyte recruitment to the sites of pathogen exposure (Kumar 2020). Nonetheless, alterations to several mechanisms initially deployed to control the infection mediate overdriven inflammation and tissue injury among septic patients.

Several cytokines listed below are overregulated during sepsis and might play a pathogenic role in this condition.

- Tumor necrosis factor alpha (TNFα) and interleukin 1 beta (IL-1β). TNFα is expressed as a membrane-bound heterotrimer and is released after shedding by a disintegrin and metalloproteinase 17 (ADAM17) in macrophages, lymphocytes, and fibroblasts. Meanwhile, IL-1β is secreted by monocytes, macrophages, and dendritic cells (DCs)(Schulte and others 2013). TNFα promotes the differentiation of macrophages (Witsell and Schook 1992), expression of intercellular adhesion molecule 1 and vascular cell adhesion molecule 1 in endothelial cells (Nakae and others 1996), and extravasation of neutrophils into tissues (Schulte and others 2013). TNFα and IL-1β are relevant in developing systemic inflammation and the accompanying coagulation disorders observed during sepsis (Schouten and others 2008). Also, they amplify the inflammatory cascade by prompting macrophages to secrete more cytokines, lipid mediators, and ROS and RNS (Cohen 2002).
- IL-6 is synthesized by macrophages, DCs, lymphocytes, endothelial cells, fibroblasts, and smooth muscle cells. It increases soluble levels of CRP, complement components, fibrinogen, and ferritin (Schulte and others 2013). Furthermore, IL-6 induces the differentiation of CD4^+^ T cells into Th17 and CD8^+^ T cells into cytotoxic T cells (Okada and others 1988; Korn and others 2009). TNFα, IL-1β, and IL-6 are considered endogenous pyrogens since they favor prostaglandin E2 synthesis and fever (Schulte and others 2013).
- CXCL8 (also named IL-8) is found at high concentrations in patients with sepsis (MERA and others 2011; Surbatovic and others 2015). CXCL8 is released by macrophages, neutrophils, eosinophils, T lymphocytes, epithelial cells, and fibroblasts, exerting a chemotactic activity on neutrophils (Bickel 1993). Hence, CXCL8 might be implicated in neutrophil-induced tissue damage, a typical lesion observed during sepsis (Shen and others 2017).
- IL-12 and interferon-gamma (IFNγ). IL-12 and IL-18 act synergistically to elicit the release of IFNγ from type 1 T helper (Th1) cells (Zhang and others 1997), but also NK cells, NKT cells, B cells, DCs, and macrophages (Nakanishi and others 2001; Nakanishi 2018). IFNγ has an important antiviral activity and stimulates M1 macrophages to produce proinflammatory cytokines, improve antigen presentation, and exert bactericidal activity (Luheshi and others 2014). Also, IFNγ antagonizes the anti-inflammatory cytokines TGB-β and IL-10 and causes fever, chills, headache, dizziness, and fatigue (Ulloa and others 1999).
- CCL2, CCL3, and CCL4 (MERA and others 2011). These chemokines attract monocytes and granulocytes to the sites of inflammation (Wolpe and others 1988; Zhang and others 1994; Menten and others 2002). Although their function is required for protective immunity against pathogens, their excessive production might worsen leukocyte recruitment and tissue damage.

Cytokines released during sepsis have profound effects on the microcirculatory system. For instance, impaired red blood cell deformability, increased blood viscosity, microvascular thrombosis, and increased nitric oxide (NO) production contribute to microcirculatory dysfunction, inadequate oxygen delivery, and tissue hypoxia (Schouten and others 2008; De Backer and others 2011). In addition, dysfunction of the vascular endothelium and loss of barrier integrity due to inflammation result in capillary leakage and interstitial edema (Goldenberg and others 2011). Likewise, altered alveolar endothelial glycocalyx induces pulmonary edema and lung injury (Maniatis and Orfanos 2008), while disruption of sinusoids is associated with hepatocellular injury and liver dysfunction (Ito and others 2006).

Persistent inflammatory responses also exacerbate the release of ROS and RNS while impairing antioxidant production, leading to oxidative stress damage. These changes alter the energy balance in the mitochondria, leading to cell death (Galley 2011). Moreover, mitochondrial damage causes the release of mitochondrial DNA and formyl peptides, which act as danger-associated molecular patterns recognized by PRRs, worsening organ injury by inducing neutrophil activation (Zhang and others 2010). In addition, some septic patients treated in intensive care units develop disseminated intravascular coagulation (Saito and others 2019).

Cytokines and chemokines activate platelets, neutrophils, and endothelial cells (Iba and Levy 2018). Vascular endothelial cells typically release NO and prostacyclin to maintain an antithrombotic state. However, activated endothelial cells become prothrombotic, producing tissue and von Willebrand factors (Iba and others 2020). Neutrophils, meanwhile, release neutrophil extracellular traps (NETs), composed of DNA, histones, and granule proteins, favoring prothrombotic activity (Camicia and others 2014).

This process causes the formation of microthrombi, which can further potentiate the inflammatory response, aggravating the microvascular dysfunction (Engelmann and Massberg 2013). Furthermore, the consumption of clotting factors generates late hemorrhagic events, which increase mortality (Greco and others 2017).

The immune system has different mechanisms to control inflammation. T regulatory (Treg) cells suppress the activity of CD4^+^ T cells, B cells, macrophages, neutrophils, and DCs (Okeke and Uzonna 2019). Decoy cytokine receptors such as IL-1 receptor antagonist (IL-1RA), IL-1 receptor type II (IL-1R2), and soluble TNFα receptors (sTNFRs) recognize specific cytokines but are unable to signal (Mantovani and others 2001). Moreover, some anti-inflammatory cytokines, such as TGF-β and IL-10, inhibit the production of proinflammatory cytokines (van der Poll and van Deventer 1999). Also, myeloid-derived-suppressor cells (MDSCs) interfere with T cell responses and regulate cytokine production from macrophages (Gabrilovich and Nagaraj 2009).

Interestingly, after the initial hyperinflammatory phase, some patients with sepsis experience a state of immunoparalysis, which is characterized by downregulation of HLA-DR on myeloid cells and apoptosis of B cells, CD4^+^ T cells, and follicular DCs (Hotchkiss and others 2001, 2002; Boomer and others 2011). Notably, the CS profile of sepsis also includes anti-inflammatory molecules such as IL-4, IL-10, and TGF-β, and decoy receptors such as IL-1RA and sTNFR (Gogos and others 2000; Surbatovic and others 2015). This immunosuppressive state is responsible for the reactivation of the infection or incidence of secondary infections, which increase sepsis's fatality (Limaye and others 2008; Torgersen and others 2009).

**FIG. 1.**
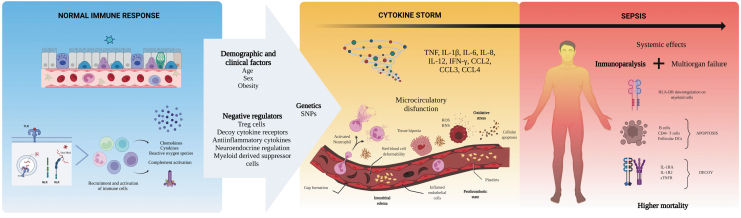
Mechanisms behind the cytokine storm of sepsis. Sepsis is an exaggerated immune reaction elicited by local or systemic infection. Individuals with this condition display elevated levels of cytokines in the circulation (hypercytokinemia), a phenomenon named “cytokine storm.” The mechanisms driving the progression from a normal immune response against a pathogen to sepsis are under investigation. Clinical and demographic features of affected persons, together with genetic factors promoting an excessive immune activation or affecting the regulatory mechanisms of the immune system, might contribute to the pathobiology of sepsis. The exuberant production of cytokines leads to harmful effects on local cells, activation, and increased permeability of the endothelium, and microthrombosis. Hypercytokinemia is also accompanied by many anti-inflammatory mechanisms that arrest immune cell functions (immunoparalysis). Together, these alterations (cytokine storm + immunoparalysis) result in the development of organ failure without clearing the infection. Understanding the pathogenesis of sepsis is crucial to approaching other severe infections such as COVID-19 and pandemic influenza. The art pieces used in this figure were modified from Biorender, licensed under a Creative Commons Attribution 3.0 Unported License. COVID-19, coronavirus disease 2019.

Cytokines also provoke a neuroinflammatory reflex through the afferent vagus nerve. Consequently, efferent vagus projections promote the secretion of acetylcholine by CD4^+^ T cells, inhibiting the excessive proinflammatory cytokine release (Rosas-Ballina and others 2011). Unfortunately, the immune system cannot return to homeostasis if the primary infection does not resolve and the regulatory mechanisms fail, inflicting more damage without clearing the infection (Fajgenbaum and June 2020). Meanwhile, persistent immunoparalysis can interfere with recovery from critical illness and increase the risk of death. Understanding the interplay of mechanisms that lead to CS and immunoparalysis during sepsis could improve our scientific approaches to other severe infections (Fig. 1).

## The CSS of Influenza

### Immunity against influenza

The influenza virus is among the primary causes of pneumonia, with 290,000–650,000 deaths and 3–5 million cases attributed to this infection annually (Shrestha and others 2011). Influenza generates a broad spectrum of symptoms, from mild to severe disease and death (Ghebrehewet and others 2016; Collaborators 2019). Type A influenza viruses are a source of annual epidemics and major pandemic outbreaks, including the H1N1 in 1918, H2N2 in 1957, H3N2 in 1968, and the most recent H1N1 in 2009 (Dunning and others 2020).

Influenza viruses belong to the Orthomyxoviridae family, and are composed of 4 genres (A to D), from which only A and B infect humans. The structure of the influenza virions has a multisegmented, negative-sense, single-strand (ss) RNA genome of 12–15 kb, with a rounded shape of 80–120 nm in diameter. Inside the capsid, the RNA and the polymerase form a viral ribonucleoprotein (vRNP) complex. The genome is segmented into 8 parts in A and B virus types (7 in C and D), which encode 8 structural proteins (PB1, PB2, PE, hemagglutinin (HA), neuraminidase (NA), M1, M2, NP), and 2 nonstructural proteins (NS1 and NEP).

HA and NA are the major glycoprotein antigens, the first facilitating the entry into the target cell, while NA mediates the release and dissemination of virions from infected cells (Krammer and others 2018). The HA binds to α 2–6 galactose and α 2–3 galactose sialic acid residues in human respiratory epithelial cells and bird gastrointestinal tract cells, respectively (Thompson and Paulson 2021). Once the virus recognizes its cell receptor, it is internalized by clathrin- and caveolin-dependent endocytosis. The vRNPs are imported to the nucleus for replication, mRNA production, and translation of novel proteins to be assembled into a new virion in the cytoplasm (Krammer and others 2018).

The innate airway defenses formed by physical barriers, mucus, phagocytic cells, cytokines, and interferon-stimulated genes (ISGs) are the first protective antiviral barrier (Martin and Frevert 2005). The respiratory epithelium secretes mucins (MUC5AC, MUC5B, MUC1, MUC 4, and MUC16), which prevent the binding of pathogens to epithelial cells (Roy and others 2014; Zanin and others 2016; Hansson 2019). The importance of mucins for defenses against influenza has been demonstrated in studies evaluating the effects of adding synthetic MUC1 molecules to epithelial cell cultures, which managed to restrain influenza viruses. Furthermore, MUC1^−/−^ mice infected with the influenza A virus display higher morbidity and mortality (McAuley and others 2017). Other molecules on the alveolar surface are the surfactant proteins A and D (SP-A and SP-D), which help viral clearance. In influenza, SP-A and SP-D bind to viral HA impeding its activity (Han and Mallampalli 2015).

The immune response against influenza initiates with the recognition of viral PAMPs and downstream signaling via host PRRs (Iwasaki and Medzhitov 2004), from which 3 pathways are essential: endosomal TLR3 and TLR7, cytoplasmatic RIG-1, and the inflammasome (Herold and others 2015). The first 2 lead to the activation of IRF3, IRF7, and NF-κB, promoting the transcription of genes encoding for cytokines, chemokines, and ISGs. RIG-1 is activated by viral ssRNA and signals by interaction with mitochondria-associated antiviral signaling proteins (Yoneyama and others 2015). Failure of RIG-1-mediated sensing of influenza viruses may lead to severe disease (Jørgensen and others 2018). Endosomal TLR3 recognizes dsRNA, and TLR7 recognizes ssRNA, activating the transcription factors NF-κB or IRF7 using signaling pathways downstream of the adapter protein myeloid differentiation factor 88 (MyD88) (Lund and others 2004; Le Goffic and others 2007).

TLR3 also interacts with the adapter Toll/IL-1R domain-containing adapter-inducing IFN-β (TRIF) and activates serine-threonine kinases (IKKɛ) and TBK1, which phosphorylates IRF3 for subsequent expression of IFN-β (Le Goffic and others 2007). The third pathway implies the formation of inflammasomes by the NLR family pyrin domain containing 3 receptor (NLRP3), which is expressed in DCs, neutrophils, macrophages, and monocytes. The detection of the viral M2 protein and a polymerase subunit (PB1) provokes the activation of this pathway. The complex is formed by NLRP3, the adapter protein apoptosis-associated speck-like protein (ASC), and procaspase-1. This complex turns on caspase-1, which cleaves the proform of IL-1β (Ichinohe and others 2010).

During influenza, cytokines and chemokines such as type I and III interferons (IFNs), IL-6, CXCL8, CCL2, CCL3, CCL4, and CCL5 are produced at the site of infection (Wareing and others 2004). Type I (IFN-α and IFN-β) and type III IFNs are critical for innate and adaptive antiviral immune responses. They interact with membrane heterodimeric receptors (IFNAR1, IFNRAR2, IFNLR1, Il-10Rβ) associated with Tyk2 and Jak1 kinases, which then phosphorylate STAT-1 and STAT-2, generating 2 activating complexes: IFN-α-activated factor (AAF) and IFN stimulated gene factor 3 (ISGF3). Already in the nucleus, these complexes bind to DNA sequences, IFNγ-activated sequence (SAG), and IFN-stimulated response element (ISRE), resulting in the stimulation of ISGs (Theofilopoulos and others 2005). Interferon-induced transmembrane (IFITM) proteins are among the host ISGs that block viral infection by frustrating cell entry at endosomes (Brass and others 2009).

As such, members of the IFITM family mediate resistance against influenza viruses (Brass and others 2009; Everitt and others 2012; Jia and others 2012; Smith and others 2013; Lanz and others 2015; Yu and others 2015; Blyth and others 2016; Meischel and others 2021; Rohaim and others 2021). Recent clinical investigations in humans have linked increased susceptibility to influenza with specific single-nucleotide polymorphisms (SNPs) in genes coding IFITM1 and IFITM3 (Everitt and others 2012; Zhang and others 2013; Allen and others 2017; Kim and others 2020, 2021).

Other cytokines and chemokines recruit neutrophils, monocytes, macrophages, NK cells, and DCs. NK cells recognize viral HA molecules through their NKp44 and NKp46 receptors and induce direct cytotoxicity or recognize infected cells through their low-affinity Fc gamma receptor FcRIIIa (CD16) that binds to IgG antibodies, leading to antibody-mediated cellular cytotoxicity (ADCC). NK cells can also release granular granzyme and perforin to induce cell lysis and secrete cytokines such as TNFα and IFNy (Jegaskanda and others 2019). Alveolar macrophages (AMs) engulf infected cells and release proinflammatory cytokines and chemokines (CCL2, CCL3, CCL4, CCL5, and TNFα) to recruit circulating monocytes, which in turn change their phenotype toward inflammatory macrophages. The latter releases CCL5, CXCL9, and CXCL10 to increase the recruitment of other leukocytes, mainly neutrophils (Latino and Gonzalez 2021).

Neutrophils migrate to the infection site and mediate phagocytosis, degranulation, the release of NETs, secretion of chemokines and cytokines (CXCL8, TNFα), and ROS production. Excessive neutrophil recruitment and degranulation destroy the lung extracellular matrix and induce epithelial apoptosis and alveolar lesions (Camp and Jonsson 2017). DCs engulf pathogens, present antigens to B and T cells, provide costimulatory signals (CD40, CD80, and CD86), and secrete cytokines (Shekhar and others 2018). The cDC2 subtype is a source of proinflammatory cytokines in the lung, whereas plasmocytoid DCs liberate large amounts of type I IFNs in response to viral infection (Thomas and others 2014).

Adaptive immunity is essential for viral clearance. CD4^+^ T cells recognize viral antigens presented by APCs on MHC-II molecules. Th1 cells produce IFNγ, IL-2, and TNFα, activating macrophages and promoting B cells to produce antibodies. Th2 lymphocytes produce IL-4, IL-5, and IL-13 and support isotype class switching in B cells (Brown and others 2006). Notably, a CD4^+^ T cell response imbalance toward the predominance of Th2 functions is detrimental to immunity against some respiratory viruses (Moran and others 1999; Pinto and others 2006). During influenza, CD8^+^ T cells are activated in the lymph nodes and migrate to the infection site, where they kill infected cells by apoptosis via Fas/FasL and perforin and granzyme degranulation (Brincks and others 2008). B lymphocytes produce neutralizing antibodies against HA and NA, which activate the complement and elicit NK cell ADCC (Stadlbauer and others 2019; Turner and others 2020b).

### Cytokine signatures of severe influenza

All the signaling pathways and cells initially deployed against influenza benefit the host by preventing viral replication and shedding; however, these mechanisms cause organ dysfunction among patients who progress to severe disease. As for sepsis, the factors that determine the switch from a protective to a harmful immune reaction are yet unclear. Perhaps host and pathogen features contribute in different proportions to establishing a CSS.

Different demographic and clinical host factors, such as sex, age, and obesity, may be involved in the susceptibility to severe influenza. Accordingly, extreme age represents a risk factor for the severity of influenza (Casalino and others 2017). In this regard, the immune system of the young can generate a strong response, whereas in the elderly, the immune response is not regulated appropriately (Aiello and others 2019). Sex was a significant prognostic factor during the 2009 pandemic since most patients hospitalized for severe disease were young women (Klein and others 2012). Finally, obese individuals with influenza display higher morbidity and mortality. High leptins and free fatty acids in obese patients might activate TLRs, monocytes, and lymphocytes to produce inflammatory cytokines (Honce and Schultz-Cherry 2019).

Genetic factors might also play a role in severe respiratory infections. Accordingly, SNPs conditioning the dysfunction of PRRs, signaling molecules, transcription factors, cytokines, chemokines, or their receptors might make an individual prone to excessive immune activation after influenza virus infection (Forbester and Humphreys 2021). Importantly, these genetic variations may lead to CS when other determinants such as the pathogen virulence, viral load at the lung, and the demographic features described above act together (de Jong and others 2006).

The immune profile observed in the circulation, bronchoalveolar lavage (BAL), and lung specimens of severely ill influenza patients is characterized by large concentrations of TNFα, IFNy, IL-1β, IL-2, IL-6, CXCL8, CCL2, CCL3, CXCL10, G-CSF, FGF, VEGF, and anti-inflammatory mediators such as TGF-β, IL-10, and IL-1RA (Meduri and others 1995; Estella 2011; Lee and others 2011; Paquette and others 2012; Bautista and others 2013; Gao and others 2013; Rendón-Ramirez and others 2015; Fiore-Gartland and others 2017; Mudd and others 2020; Choreño-Parra and others 2021a; Reynolds and others 2021; Xie and others 2021). These cytokines generate lung damage when overproduced by different mechanisms, some of which were mentioned above. Their damaging properties cannot be experimentally tested in humans, but animal models have proven that these cytokines mediate the morbidity and mortality of influenza.

An important cytokine for antiviral defenses that plays a pathogenic role during severe influenza is IFNy, mainly produced by adaptive Th1 cells. In mice with influenza A (H1N1), antibody neutralization of IFNy reduces lung tissue inflammation and BAL cytokine levels, and improves survival (Liu and others 2021). IL-1β is another cytokine harmful during influenza. Indeed, mice with genetic deficiency of the inflammasome complex NLRP3/ASC/caspase-1 are less susceptible to lung inflammation and mortality by viral H7N9 influenza infection (Ren and others 2017). Finally, IL-6 favors neutrophil recruitment and B cell differentiation. However, its excessive secretion is linked to severe illness. Importantly, inhibition of IL-6 by the suppressor of cytokine signaling 3 (SOCS-3) improves influenza outcomes by reducing inflammation in mice (Liu and others 2019).

The data summarized above indicate that cytokines produced by strong immune responses cause severe manifestations of influenza. Although the mechanisms of predisposition to the CSS are not well defined, lessons from the study of sepsis and influenza pathogenesis might be important to approach other infections such as COVID-19.

## The CSS of COVID-19

### Immunity against SARS-CoV-2

SARS-CoV-2 is an enveloped, positive-sense ssRNA virus of the Coronaviridae family, genus Beta coronavirus, including SARS-CoV and MERS-CoV (Wu and others 2020b; Zhou and others 2020a). Its genome contains 14 major open reading frames (ORFs) coding for nonstructural, accessory, and structural proteins. The ORFs 10 and 11 encode for 4 structural proteins named spike (S), envelope (E), membrane (M), and nucleocapsid (N) (Lim and others 2016). The S protein attaches to the cellular receptor angiotensin-converting enzyme metallopeptidase 2 (ACE2), thus determining infectivity and viral tropism (Li 2016). This enzyme is found in the lungs, blood vessels, small intestine, and kidney, among other organs, suggesting alternative transmission routes and explaining the multiorgan damage observed in critically ill COVID-19 patients (Hamming and others 2004). CD147 has been proposed as another SARS CoV-2 receptor (Wang and others 2020a).

Meanwhile, protein E is a viroporin that participates in releasing newly assembled viral particles. Studies in SARS-CoV have shown that the deletion of protein E does not affect viral production but reduces virion maturation and viral load (Schoeman and Fielding 2019). In addition, the E protein has a lower mutational rate than the S protein, making it a candidate target for vaccines (Sarkar and Saha 2020). Protein M is capable of binding to all the other structural proteins. Despite its undefined function, its binding to the N protein allows its stabilization and, therefore, indirectly participates in the viral genome assembly. Also, the structure of M protein suggests a potential sugar transporter function such as the sugar transporter SemiSWEET protein found in prokaryotic cells (Thomas 2020).

Finally, protein N is among the most abundant and immunogenic SARS-CoV-2 proteins that participate in the transcription and assembly of the viral genome and immune evasion (Cubuk and others 2021).

The most common SARS CoV-2 infection route is the respiratory system. In this study, the S protein binds to ACE2 in the plasma membrane of pneumocytes. This protein owns 2 functional domains: the S1 domain contains the receptor-binding domain, which attaches to ACE2, whereas the S2 domain mediates the fusion of the viral and host cell membranes (Walls and others 2020). For effective infection, the host transmembrane serine protease-2 (TMPRSS-2) cleaves to the S2 subunit of the protein (Glowacka and others 2011; Matsuyama and others 2020). Other host proteases such as furin, TMPRSS4, and cathepsin L also activate the S2 protein (Ou and others 2020; Zang and others 2020). Recently, neuropilin-1 has been identified as another host factor facilitating SARS-CoV-2 infectivity (Hoffmann and others 2020; Matsuyama and others 2020).

The entry mechanisms of coronaviruses are unclear. Initially, researchers thought that SARS-CoV entry was by the direct release of viral particles into the cells after complete membrane fusion. However, SARS-CoV and SARS-CoV-2 also utilize clathrin-dependent endocytosis (Wang and others 2008; Bayati and others 2021).

As for influenza viruses, mucins and collectins at mucosal respiratory barriers play an essential role against SARS-CoV-2 (Bose and others 2021). Accordingly, increased MUC1 and MUCl5AC have been observed in the sputum of patients with COVID-19 (Lu and others 2021). Also, animal studies demonstrated that MUC4 protects the female, but not male mice from SARS-CoV-2 (Plante and others 2020). Surfactant proteins with immune properties may also participate in airway antiviral defenses. Indeed, elevated levels of SP-D have been observed in the blood of patients with severe COVID-19 (Tong and others 2021), suggesting a leakage from the airway due to alveolar damage.

This blood translocation of SP-D might be less severe than in influenza (Choreño-Parra and others 2021b), but could be used as a lung injury readout. Interestingly, recombinant fragments of SP-D bind and neutralize the viral S protein functions (Hsieh and others 2021), while mannose-binding lectin recognizes glycosylated sites of the S protein neutralizing SARS-CoV-2 infectivity (Stravalaci and others 2022).

The PRRs that recognize SARS-CoV-2 and initiate the immune responses remain obscure. As this virus is genetically related to SARS-CoV, both viruses may share mechanisms of infection. For instance, SARS-CoV is recognized by TLR3 and TLR4, which induce MyD88 and TRIF pathways (Sheahan and others 2008; Totura and others 2015). TLR4 has been proposed to detect SARS-CoV-2 (Aboudounya and Heads 2021), but complementary evidence is required. TLR2 also recognizes the E protein of SARS-CoV-2 and activates MyD88 signaling to initiate the production of IL-1β, IL-6, TNFα, IFNy, and CXCL10 (Zheng and others 2021). Finally, the viral RNA sensors TLR3 and TLR7 promote the release of type I and type III IFNs, IL-1β, IL-4, IL-6, and IFNy, through IFR3 and NFκB pathways (Bortolotti and others 2021). In addition, SARS-CoV triggers the bioactivation of IL-1β through NLRP3 inflammasomes (Shi and others 2019).

Similarly, SARS-CoV-2 N protein promotes NLRP3 inflammasome activation (Pan and others 2021), explaining the high levels of IL-1β observed in COVID-19 patients (Rodrigues and others 2021).

Type I interferons and ISGs are strongly upregulated during SARS-CoV-2 infection (Lee and others 2020; Mantlo and others 2020; Rosa and others 2021). Indeed, higher levels of IFN-α, IFN-β, IL-2, and IL-12 are distinctive features of asymptomatic and mild as opposed to severe COVID-19 (Masood and others 2021; Tjan and others 2021). Type I IFNs reduce the infectivity of SARS-CoV-2 *in vitro* (Mantlo and others 2020), whereas IL-2 and IL-12 might contribute to protection by stimulating T and B cell growth and differentiation. Among other ISGs transcribed during COVID-19, IFITMs might be necessary, and some studies have linked the prevalence of SNPs affecting *IFITM3* to COVID-19 susceptibility (Gómez and others 2021; Schönfelder and others 2021).

The initial recognition of SARS-CoV-2 also promotes chemotaxis. Noticeably, in patients with severe COVID-19, an ample range of immune cell subtypes are depleted from the circulation, including monocytes, DCs, CD4^+^ T cells, CD8^+^ T cells, B cells, and NK cells. This phenomenon is accompanied by peripheral neutrophilia and intense leukocyte infiltration of the lung (Liao and others 2020; Merad 2020; Wang and others 2020b; Wang and others 2020; Xu and others 2020; Zheng and others 2020), suggesting the potential participation of specific immune cell subsets in defenses against SARS-CoV-2.

Neutrophils are the principal cells recruited to the lung of COVID-19 patients. These cells degranulate, phagocyte the virus, and liberate NETs (Wu and others 2020a; Reusch and others 2021; Rosa and others 2021). However, their exuberant recruitment and function lead to tissue damage and a readout of COVID-19 severity (Hernández-Cárdenas and others 2021). In the lung, distinct AM subpopulations engulf SARS-CoV-2 to initiate the local immune response. However, the virus can escape from these cells and evade innate immunity (Dalskov and others 2020; Lv and others 2021). Then, attracted by chemokines such as CCL2, CCL3, and CCL4, monocytes and macrophages migrate to contribute to antiviral defenses by phagocytosis of virus and infected cells and cytokine production to amplify the response. Nevertheless, intense recruitment of inflammatory monocytes causes excessive production of proinflammatory molecules and neutrophil infiltration, which might lead to injury (Merad 2020; Vanderbeke and others 2021).

Populations of adaptive NK cells with enhanced cytotoxic functions may also participate in antiviral defenses, as indicated by studies demonstrating increased circulation of NKG2C^+^ memory-like NK cells in patients with COVID-19 (Maucourant and others 2020). Interestingly, deleting mutations in genes coding for NKG2C and its ligand HLA-E and dysfunction of NK cells are associated with a higher risk of severe COVID-19 (Krämer and others 2021; Vietzen and others 2021). Moreover, NK cells from severe COVID-19 patients express PD-1, a marker of functional exhaustion (Wilk and others 2020).

The initiation of adaptive immune responses is pivotal for infection control, viral clearance, and short-term protection against reinfection, as demonstrated in studies of COVID-19 vaccines (Folegatti and others 2020; Ewer and others 2021; Levin and others 2021; Lustig and others 2021). In this regard, vaccination and natural infection with SARS-CoV-2 elicit germinal center (GC) reactions at secondary lymphoid organs where B cells activate and differentiate into plasma cells that produce neutralizing antibodies with the cooperation of follicular T helper cells (Shaan Lakshmanappa and others 2021; Turner and others 2021a, 2021b). Significantly, the failure in follicular T cell activation and promotion of GCs is associated with severe COVID-19 (Kaneko and others 2020).

**FIG. 2.**
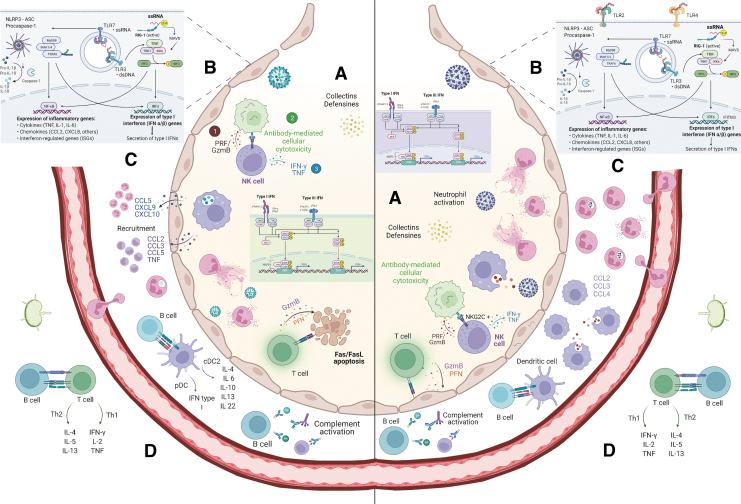
Immune mechanisms implicated in the defense against SARS-CoV-2 and influenza. **(A)** Innate humoral factors present in the lumen of the lower airways block viruses before they attach the underlying epithelium. Mucins and surfactant proteins A and D are important for host defenses against influenza virus and SARS-CoV-2. Mannose-binding lectin has also shown to neutralize SARS-CoV-2. **(B)** The innate immune response against these viruses begins with recognizing PAMPs by host PRRs. TLR3, TLR7, RIG-1, and the NLRP3 inflammasome participate in the early recognition of influenza and SARS-CoV-2, eliciting the production of cytokines, chemokines, and interferons. TLR2 and TLR4 may also participate in the defense against SARS-CoV-2, but the evidence is still scarce. **(C)** The innate phase of the immune response against influenza and SARS-CoV-2 comprehends an ample range of mechanisms, including the chemotaxis of monocytes, neutrophils, other granulocytes, and neutrophil degranulation and NETosis, phagocytosis of viral particles and infected cells, and cytotoxicity by NK cells. Some populations of NK cells with adaptive properties (NKG2C^+^) might also expand during COVID-19. **(D)** Dendritic cells link innate and adaptive immunity by presenting antigens at local lymph nodes and secreting cytokines that shape the functional fate of B and T cells. B cells produce neutralizing antibodies that mediate complement activation and antibody-dependent cellular cytotoxicity. CD8^+^ T cells kill infected cells by perforin and granzyme degranulation or via the Fas/FasL signaling pathway. CD4^+^ T cells produce cytokines to orchestrate all the other mechanisms described. A balance between Th1 and Th2 responses might be crucial for antiviral immunity. The art pieces used in this figure were modified from Biorender, licensed under a Creative Commons Attribution 3.0 Unported License. NK, natural killer; NLRP3, NLR family pyrin domain containing 3 receptor; RIG-1, retinoic-acid-inducible gene 1; SARS-CoV-2, severe acute respiratory syndrome coronavirus 2; TLR, Toll-like receptor.

Finally, cytotoxic CD8^+^ T cells also participate in SARS-CoV-2 elimination and may be particularly important against novel coronavirus variants with improved evasiveness of humoral immunity (Naranbhai and others 2022). Figure 2 summarizes the current knowledge about defensive immune mechanisms against SARS-CoV-2 and how they compare with immunity versus influenza.

### Cytokine signatures during severe COVID-19

A better understanding of the immune factors implicated in the pathophysiology of COVID-19 is crucial to guiding the development of novel vaccines and immunotherapeutics. Unfortunately, what we comprehend about severe COVID-19 is contradictory. First, the immune response against SARS-CoV-2 is overregulated. Nevertheless, this excessive reaction is not protective and instead causes tissue injury. Patients with severe COVID-19 display elevated levels of proinflammatory and anti-inflammatory cytokines, chemokines, and growth factors, accompanied by increased neutrophil counts, lymphopenia, and depletion of different cellular subsets in the circulation, as mentioned above.

The factors aiding the transition from a protective to a dysregulated immune response are elusive, but there is much interest in identifying risk factors associated with worse clinical outcomes in COVID-19. Again, clinical variables such as age and sex are important. Aging is associated with declined immunity and confers higher odds of death in patients with COVID-19 (Costagliola and others 2021). For instance, elderly humans and primates display increased neutrophilic inflammation than young individuals after SARS-CoV-2 infection (Rosa and others 2021). Remarkably, the male gender is disproportionally associated with worse outcomes in COVID-19. The higher expression and distinct tissue distribution of ACE2 and the possible immune alterations common in males might explain this discrepancy (Peckham and others 2020). The ample spectrum of immune deficiencies induced by metabolic disruption might account for the higher risk for severe COVID-19 in obese and in diabetic patients (Holly and others 2020).

In contrast, host genetic factors determining higher susceptibility to CS are poorly recognized since recent studies have only identified genetic abnormalities conditioning immune dysfunction, but not hyperinflammation (Forbester and Humphreys 2021; Velavan and others 2021).

Profiling immune mediators in severe COVID-19 patients have revealed low concentrations of type I interferons (Hadjadj and others 2020; Masood and others 2021), and elevated levels of TNFα, IFNy, IL-1β, IL-1RA, IL-4, IL-6, IL-7, CXCL8, IL-9, IL-17A, CCL2, CCL3, CCL4, CCL5, CCL7, CCL8, CCL11, CXCL9, CXCL10, G-CSF, GM-CSF, PDGF, FGF, and VEGF (Chen and others 2020; Han and others 2020; Huang and others 2020; Kong and others 2020; Lucas and others 2020; Remy and others 2020; Wan and others 2020; Yang and others 2020; Zhu and others 2020b; Reynolds and others 2021; Sims and others 2021). From these, CXCL10, a downstream IFNy effector molecule, shows a strong correlation with disease severity (Yang and others 2020) and is highly detectable in the airways of COVID-19 patients (Reynolds and others 2021).

This chemokine, together with CXCL8, recruits neutrophils after binding to CXCR3 (Ichikawa and others 2013), thus exacerbating neutrophil-induced lung damage (Wilk and others 2020; Rosa and others 2021; Vanderbeke and others 2021). CXCR3 is also expressed on macrophages, activated Th1 cells, B lymphocytes, NK cells, and DCs (Groom and Luster 2011). Hence, CXCL10 might be a suitable target to reduce lung inflammation in COVID-19 patients. Meanwhile, the role of IL-9, the classical cytokine of Th9 cells, is unknown in COVID-19.

However, the magnitude of Th9 responses has been associated with the severity of respiratory syncytial virus infection (Pinto and others 2006). CCL5 is chemotactic for T cells, eosinophils, and basophils expressing the receptor CCR5, and its blockade reduces inflammation and viremia in critically ill COVID-19 patients (Patterson and others 2020), whereas CCL7 attracts monocytes and eosinophils and is associated with the severity of the disease (Yang and others 2020). GM-CSF is a myeloid cell growth factor and proinflammatory signal instructing macrophages to amplify cytokine cascades. GM-CSF is secreted by macrophages, T cells, mast cells, NK cells, endothelial cells, and fibroblasts and might be a pivotal driver of lung inflammation in severe COVID-19 (Leavis and others 2022). Notably, the GM-CSF blockade improves clinical symptoms and survival in patients with COVID-19 (De Luca and others 2020).

Intriguingly, the CS of severe COVID-19 is also accompanied by functional impairment of myeloid cells and lymphocytes (Remy and others 2020), resembling the immunoparalysis that accompanies hypercytokinemia in sepsis. Impaired type I IFN production might advocate this immunocompromised state (Hadjadj and others 2020; Masood and others 2021). Also, mixed signals might provide immune cells with confounding instructions making them functionally impaired. Indeed, different patterns of cytokine and chemokine combinations in COVID-19 patients can be identified according to their disease trajectory, showing that some individuals with the worse outcomes display mixed polyfunctional cytokine signatures (Lucas and others 2020). Furthermore, the anti-inflammatory cytokines TGF-β and IL-10 have been detected in high concentrations during SARS-CoV-2 infection and might suppress immune cell functions (Han and others 2020; Wan and others 2020; Ferreira-Gomes and others 2021).

## Face-to-Face: Immune Profiles of Severe Influenza and COVID-19

As remarked in the article, the study of sepsis and severe influenza has provided reference knowledge to face COVID-19. Currently, it is accepted that the clinical landscape of COVID-19 mirrors other infectious CSS in many aspects. This assumption relies on literature reviews and retrospective studies highlighting similarities between patients infected with SARS-CoV-2 and influenza (Jiang and others 2020; Tang and others 2020). Indeed, several symptoms are shared by both infections, probably due to a similar pathophysiology. Nonetheless, detailed analysis reveals that some clinical features distinguish each disease, perhaps because of molecular properties, tropism determinants, and virulence factors of each virus. Table 1 summarizes the main similarities and differences in viral characteristics and clinical findings of COVID-19 and influenza. The rest of this section focuses on comparing the CSS of both diseases.

**Table 1.** Viral and Clinical Characteristics of COVID-19 and Influenza

Characteristic	Influenza	COVID-19
Virus identification	1918, United States	2019, China
Virus family	Orthomyxoviridae	Coronaviridae
Viral nucleic acid	Single-stranded RNA (negative sense) 13.5 kb	Single-stranded RNA (positive sense) 26–32 kb
Animal reservoirs	Birds, pigs	Bats? Pangolin?
Mechanism of transmission	Inhalation	Inhalation
Incubation period	2 days	2–14 days
R0	2	2.5
Genome variation mechanism	Reassort and rearrange	Point mutations
Viral proteins of interest	HA, NA	*S*, E, M
Host receptor	α 2,6 sialic acids	ACE2
Tropism	Respiratory tract epithelium	Multiple organs
Frequent symptoms	Fever, dyspnea, cough	Fever, dyspnea, cough
Distinctive manifestations	High fever, headache, fatigue, myalgia, sore throat, cough, eye symptoms	Nonproductive cough, fatigue, myalgia, gastrointestinal symptoms, anosmia, dysgeusia
Radiological findings	Multilobe consolidations	Ground-glass opacities
High-risk populations	Elderly, pregnant women, people with respiratory diseases, hypertension, coronary heart disease, diabetes, kidney disease, liver disease, malignancy	Elderly, people with respiratory diseases, obesity, hypertension, coronary heart disease, diabetes, malignancy
Need for hospitalization	5.6%	20%
Need for intubation	4.8%	10%–15%
Mortality	0.13%–1.36%	1.40%–3.67%
Sequela	20%–30%	25%–40%

ACE2, angiotensin-converting enzyme metallopeptidase 2; COVID-19, coronavirus disease 2019; HA, hemagglutinin; NA, neuraminidase.

### The potential behind similarities

**FIG. 3.**
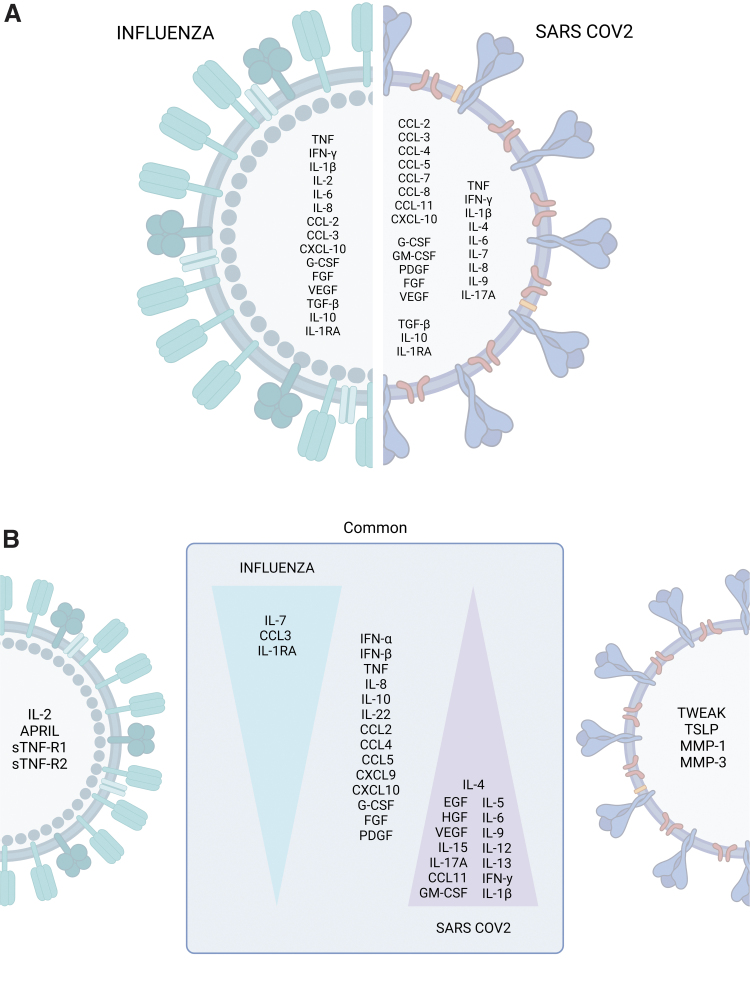
The cytokine storm profiles of pandemic influenza and COVID-19. **(A)** Cytokines, chemokines, and growth factors commonly or differentially elevated during severe influenza and COVID-19 were identified by retrospective analysis of independent studies. **(B)** Immune profiles distinguishing influenza from COVID-19 identified by parallel comparisons. The art pieces used in this figure were modified from Biorender, licensed under a Creative Commons Attribution 3.0 Unported License.

Using the data summarized here, we can conclude that the CSS of severe COVID-19 coincides with influenza, indicating common pathological mechanisms that could be exploited for therapeutic purposes. Certainly, both viruses are recognized by similar PRRs, trigger the same signaling pathways, and require similar innate and adaptive immune components for protection. As shown in panel A of Fig. 3, the CS of severe influenza and COVID-19 concurs in elevated PRR- and inflammasome-induced cytokines, such as TNFα, IL-1β, and IL-6, revealing a persistent innate inflammatory reaction that is detrimental to the host. Hypothetically, targeting these molecules could reduce their vascular and immunological effects, which are key in the pathogenesis of sepsis, calming inflammation and allowing the lung and extrapulmonary organs to restore homeostasis.

To this matter, broad transcriptional suppression of innate inflammatory genes might be achieved using corticosteroids. For instance, dexamethasone effectively reduces the morbidity of patients with severe COVID-19 (Group and others 2021). This drug has minor mineralocorticoid effects and reduces inflammation by enhancing the deacetylation of the histones that regulate cytokine gene expression (Barnes 2006). Conversely, corticosteroids increase the rates of coinfection and death in patients with influenza (Zhou and others 2020b), although recent trials indicate a potential benefit for survival (Villar and others 2020).

Direct blockade of TNFα (infliximab), IL-1R (anakinra, canakinumab), IL-6 (siltuximab, olokizumab), and IL-6R (tocilizumab, sarilumab, levilimab) is being tested in clinical trials, showing promising benefits by reducing symptomatic burden, need for invasive respiratory support, and death, thus warranting further investigation, as revised elsewhere (Pum and others 2021). TNFα antagonism would warrant additional research about the timing of treatment administration since TNFα is potentially protective during the early stages of influenza and SARS-CoV-2 infection. Some observations of individuals already taking anti-TNFα therapies that showed milder symptoms after getting positive for COVID-19 might dissipate this concern (Abdullah and others 2020). On the contrary, tocilizumab is among the immunotherapies most extensively evaluated in COVID-19. By the time SARS-CoV-2 emerged, this agent had already proven safety and efficacy against other CSS (Yokota and others 2008; Kotch and others 2019), facilitating its rapid reallocation.

Although most studies show clinical benefits, data supporting tocilizumab lack reproducibility (Price and others 2020), perhaps because of methodological heterogeneity of clinical trials. Meanwhile, there is little evidence regarding the use of tocilizumab in patients with influenza. Two small studies have shown that patients previously receiving this treatment display milder symptoms of infection (Kawada and others 2013), and tocilizumab does not affect antibody responses against influenza vaccines (Mori and others 2012), supporting that tocilizumab could be safely used for influenza patients. A relevant aspect to consider for anti-IL-6 immunotherapy of infectious CSS is the effects of IL-6 on adaptive immunity and T cell differentiation, which vary depending on the concentration of other cytokines in the milieu (Martinez-Sanchez and others 2018), and, if altered, could lead to detrimental effects.

Hence, tocilizumab administration should be guided not only by IL-6 concentrations but also by each patient's cytokine and immune cell profile. This premise might apply to other immunotherapeutics as well.

Remarkably, severe influenza and COVID-19 also converge in elevated levels of chemotactic (CXCL8, CCL2, CCL3, and CXCL10) and activating molecules (G-CSF) acting on monocytes and neutrophils. As mentioned above, a range of monocyte and neutrophil subsets with inflammatory and degranulating phenotypes mediate lung inflammation and disease progression in influenza and COVID-19 (Turner and others 2020a; Wilk and others 2020; Rosa and others 2021; Vanderbeke and others 2021). Hence, disruption of these chemotactic axes is also an attractive therapeutic approach. Currently, only a clinical trial is evaluating the effect of an anti-CXCL8 antibody for the treatment of COVID-19 (NCT04347226), but no results have been posted. Therefore, more research on the antagonism of CXCL8, CCL2, CCL3, and CXCL10 in influenza and COVID-19 is required.

Interestingly, innovative approaches to disrupt chemotaxis using molecular engineered decoy CCL2 and CXCL8 proteins deserve additional evaluation (Adage and others 2015a, 2015b; Roblek and others 2016). Despite this, inhibiting chemotaxis could require administering various agents at a time because of the considerable redundancy of the human chemokine axes. The side effects of CXCL10 blockade in immune protection against influenza and COVID19 should also be tested due to the functions of this chemokine in mobilizing T cells. Similarly, the therapeutic potential behind antagonizing G-CSF has not been addressed, but recent observations of detrimental consequences of the opposite approach (G-CSF administration) in COVID-19 patients are proof of the concept (Taha and others 2020; Sereno and others 2021).

Historically, IFNy has been considered the dominant protective mechanism against intracellular pathogens. In contrast, in the light of fresh visions, IFNy-mediated Th1 responses are highly destructive backup responses only deployed when innate defenses fail in clearing infections (Matzinger and Kamala 2011). High levels of IFNy in patients with severe but not mild-to-moderate influenza and COVID-19 reinforce this idea. Emapalumab, a monoclonal antibody against IFNy, is safe and effective in reducing the CSS of primary HLH (Locatelli and others 2020), and is currently under evaluation for CSS of severe COVID-19 (NCT04324021).

Immune mediators with strong effects on the endothelium, such as FGF and VEGF, are also potential objectives of immunotherapy to reduce morbidity derived from microvascular abnormalities during severe influenza and COVID-19. VEGF is of particular interest as this marker correlates with acute kidney injury development and progression to severe disease in influenza and COVID-19 patients, respectively (Bautista and others 2013; Kong and others 2020). VEGF inhibition with bevacizumab is used harmlessly to reduce angiogenesis associated with lung cancer and ocular disorders (Lauro and others 2014; Afarid and others 2018).

A small phase 2 study has shown some clinical potential of bevacizumab in critically ill patients with COVID-19 (Pang and others 2021), but the evidence is still scarce. Lastly, the interruption of the effects of elevated TGF-β, IL-10, and IL-1RA levels might help overcome the immune cell exhaustion and immunosuppression that accompany the CS of these infections. However, extensive experimentation is required before clinical applications are attempted since molecules such as TGF-β and IL-10 have concentration-dependent effector and regulatory properties, such as promoting IgA production in epithelia.

### Influenza versus COVID-19: targeting differences

Beyond the parallelisms between influenza and COVID-19 aforesaid, a compilation of retrospective data from independent studies indicate that IL-2 increases only during severe influenza, whereas high concentrations of IL-4, IL-7, IL-9, IL-17A, CCL4, CCL5, CCL7, CCL8, CCL11, GM-CSF, and PDGF are exclusive features of severe COVID-19 (Fig. 3A). So, what is clear is the ample and polyfunctional CS profile elicited by SARS-CoV-2 but not the influenza virus. Nevertheless, to identify distinctive CS components of COVID-19 and influenza, the problem with retrospective comparisons is the risk of biased conclusions due to differences in the genetic background, sociocultural characteristics, technological infrastructure, and research logistics in different regions.

Another caveat is that molecules identified by this approach are observed in severe but not mild-to-moderate forms of each disease, without side-to-side contrasting of both infections. Furthermore, some cytokines could be measured independently in one disease group but not the other. Hence, parallel analyses in geographical settings with similar resources would provide a better perspective. Surprisingly, although the emergence of SARS-CoV-2 occurred near the peak of the 2019–2020 influenza season (Poyiadji and others 2020; Zhu and others 2020a), only a few comparative studies have been conducted (Lee and others 2020; Mudd and others 2020; Vaz de Paula and others 2020; Choreño-Parra and others 2021a, 2021b, 2021c; Guo and others 2021; Olbei and others 2021; Reynolds and others 2021), which has also been difficulted by a reduction in the circulation of influenza viruses following the COVID-19 pandemic.

As shown in Fig. 3B, data from parallel comparisons exhibit a broad spectrum of elevated molecules in both diseases. From these, several cytokines with antiviral (IFN-α, IFN-β), inflammatory (TNFα, IL-12, IL-22), regulatory (IL-10), chemoattractant (CXCL8, CCL2, CCL4, CCL5, CXCL9, CXCL10), angiogenic (FGF, PDGF, PDGF), and growth factor (G-CSF, FGF, PDGF) properties are constantly upregulated in severe influenza and COVID-19. These findings provide further rationale for immunotherapy directed to regulate innate inflammation, monocyte/neutrophil chemotaxis, and vasoactive cytokines to reduce the morbidity associated with these CSSs.

The second category of molecules is only elevated in one disease but not the other. For instance, severe influenza differs from COVID-19 by higher levels of IL-2, APRIL, sTNF-R1, sTNF-R2, SP-D, and CXCL17. These mediators exert important functions to sustain protective immunity. IL-2 and APRIL support T cell and plasma cell survival (Benson and others 2008), respectively, while sTNF-R1/R2 are decoy receptors that balance the destructive capacity of TNFα (Pennica and others 1993). CXCL17 is a mucosal chemokine expressed in the respiratory tract that mediates myeloid-cell recruitment and anti-inflammatory activities (Choreno-Parra and others 2020). The elevated CXCL17 levels observed only in severe influenza patients might indicate that they have more regulatory mechanisms to minimize tissue damage than individuals with COVID-19. Thus, immunotherapy against these factors might not be suitable, but the observations reveal important differences in the pathogenesis of influenza.

Conversely, TWEAK, TSLP, MMP-1, and MMP-3 are upregulated only in COVID-19. TWEAK is an amplifier of inflammation that stimulates the further secretion of IL-6, CXCL8, CXCL10, and MMP-1 (Saas and others 2000; Chicheportiche and others 2002). Therapeutic targeting of TWEAK might calm inflammation and reduce the morbidity of COVID-19. Since TWEAK might promote cancer cell survival, a monoclonal antibody developed to block the TWEAK receptor (enabatuzumab) is being tested clinically in cancer trials (Lam and others 2018), although it has possible hepatotoxic effects. TSLP is a promoter of allergic inflammation and Th2 responses (Ito and others 2012). The matrix metalloproteinases MMP-1 and MMP-3 are implicated in tissue damage underlying other lung diseases (D'Armiento and others 1992; Dahlen and others 1999; Greenlee and others 2007), placing them as potential therapeutic objectives to reduce lung injury in COVID-19. Nonetheless, validation studies are required to demonstrate a link between TWEAK, TLSP, MMP-1, and MMP-3 and severe COVID-19.

A third cytokine cluster includes molecules found in severe influenza and COVID-19, but with higher frequency and concentrations in one CSS than its counterpart. Interestingly, the profile of this cluster in COVID-19 again shows a mixed Th1/Th2/Th9/Th17 response, together with innate cytokines (IL-1β, IL-6), eosinophil chemokines (CCL11), growth factors, and vasoactive molecules (GM-CSF, HGF, EGF, VEGF). Hence, the lack of balance of the effector response might be another determinant of the host defensive collapse observed in some critical COVID-19 patients. Specifically, the Th2 component of this response might inhibit antiviral responses in specific subgroups of patients and generate interstitial infiltrates of neutrophils, eosinophils, and type 2 innate lymphoid cells (ILC2s), mediating lung inflammation and tissue damage. In fact, evidence exists that Th2 mediators and eosinophilia are associated with worse outcomes in a subset of individuals with severe COVID-19 (Fraissé and others 2020; Lucas and others 2020).

Furthermore, histopathological analyses of postmortem lung specimens have confirmed that COVID-19 differs from influenza by a robust Th2 response that accompanies local Th1 and Th17 inflammation in some fatal cases (Vaz de Paula and others 2020; Choreño-Parra and others 2021a). These deleterious effects of Th2 responses could also initiate pathogenic processes that favor the progression to pulmonary fibrosis, as observed in several severe COVID-19 patients discharged from hospitals (Mo and others 2020).

Considering the evidence, we propose that the optimal immune therapeutics for COVID-19 should not only block specific immune signaling pathways associated with hyperinflammation but also reestablish a convenient immune balance that promotes protective immunity in the specific subgroup of patients who display polyfunctional cytokine production. For this purpose, some cytokines could be targeted. For instance, monoclonal antibodies against IL-4 (dupilumab) have been used in patients with atopic dermatitis and COVID-19 without increasing the risk of severe complications and even apparently reducing respiratory symptoms (Caroppo and others 2020; Carugno and others 2020; Ferrucci and others 2020; Ungar and others 2022). IL-9 and TSLP could be other targets to inhibit Th2 responses in COVID-19 patients, as these molecules promote allergic inflammation (Temann and others 2002; Ito and others 2012; Koch and others 2017). Monoclonal antibodies against IL-9 (MEDI-528) and TSLP (tezepelumab) are currently in clinical trials for asthma.

Although MEDI-528 inhibits several aspects of the immunopathology of asthma in mice, clinical data are yet scarce (Gong and others 2017). Conversely, tezepelumab improves lung function and reduces eosinophilia and exacerbations in patients with uncontrolled asthma (Menzies-Gow and others 2021). Hence, future studies should assess whether tezepelumab could improve outcomes in COVID-19.

## Concluding Remarks

The data summarized in this article reveal important similarities and differences in the immune profile of severe influenza and COVID-19. These diseases display increased levels of cytokines with anti-viral (IFN-α, IFN-β), inflammatory (TNFα, IL-12, IL-22), regulatory (IL-10), chemoattractant (CXCL8, CCL2, CCL4, CCL5, CXCL9, CXCL10), angiogenic (FGF, PDGF, PDGF), and growth factor (G-CSF, FGF, PDGF) properties. Hence, pathogenic mechanisms such as excessive innate immune activation, monocyte/neutrophil chemotaxis, and microvascular dysfunction might be important during the 2 diseases. Conversely, discrepancies in the immune signature of these infections include higher levels of Th1 cytokines along with IL-2, APRIL, sTNF-R1, sTNF-R2, SP-D, and CXCL17 in severe influenza patients, with COVID-19 displaying a polyfunctional Th1/Th2/Th17 immune activation profile in some patients with severe manifestations. Hence, reestablishing a balanced immune reaction might be a good objective for host-directed therapies directed to certain subgroups of COVID-19 patients.

Nonetheless, additional research is warranted to validate these immune profiles and clarify the best timing for administering specific immunotherapies according to the cytokine dynamics of these infections.

## References

[B1] Abdullah A, Neurath MF, Atreya R. 2020. Mild COVID-19 symptoms in an infliximab-treated ulcerative colitis patient: can ongoing anti-TNF therapy protect against the viral hyperinflammatory response and avoid aggravated outcomes? Visceral Med 36(4):338–342. 10.1159/000508740 PMC7316657 32999889

[B2] Aboudounya MM, Heads RJ. 2021. COVID-19 and Toll-Like Receptor 4 (TLR4): SARS-CoV-2 May Bind and Activate TLR4 to Increase ACE2 expression, facilitating entry and causing hyperinflammation. Mediat Inflamm 2021:8874339. 10.1155/2021/8874339 PMC7811571 33505220

[B3] Adage T, Del Bene F, Fiorentini F, Doornbos RP, Zankl C, Bartley MR, Kungl AJ. 2015a. PA401, a novel CXCL8-based biologic therapeutic with increased glycosaminoglycan binding, reduces bronchoalveolar lavage neutrophils and systemic inflammatory markers in a murine model of LPS-induced lung inflammation. Cytokine 76(2):433–441. 26303011 10.1016/j.cyto.2015.08.006

[B4] Adage T, Konya V, Weber C, Strutzmann E, Fuchs T, Zankl C, Gerlza T, Jeremic D, Heinemann A, Kungl AJ. 2015b. Targeting glycosaminoglycans in the lung by an engineered CXCL8 as a novel therapeutic approach to lung inflammation. Eur J Pharmacol 748:83–92. 25554213 10.1016/j.ejphar.2014.12.019

[B5] Afarid M, Sadegi Sarvestani A, Rahat F, Azimi A. 2018. Intravitreal injection of bevacizumab: review of our previous experience. Iranian J Pharm Res 17(3):1093–1098. PMC6094424 30127831

[B6] Aiello A, Farzaneh F, Candore G, Caruso C, Davinelli S, Gambino CM, Ligotti ME, Zareian N, Accardi G. 2019. Immunosenescence and its hallmarks: how to oppose aging strategically? a review of potential options for therapeutic intervention. Front Immunol 10:2247. 31608061 10.3389/fimmu.2019.02247 PMC6773825

[B7] Al-Samkari H, Berliner N. 2018. Hemophagocytic lymphohistiocytosis. Annu Rev Pathol 13:27–49. 28934563 10.1146/annurev-pathol-020117-043625

[B8] Allen EK, Randolph AG, Bhangale T, Dogra P, Ohlson M, Oshansky CM, Zamora AE, Shannon JP, Finkelstein D, Dressen A, DeVincenzo J, Caniza M, Youngblood B, Rosenberger CM, Thomas PG. 2017. SNP-mediated disruption of CTCF binding at the IFITM3 promoter is associated with risk of severe influenza in humans. Nat Med 23(8):975–983. 28714988 10.1038/nm.4370 PMC5702558

[B9] Barnes PJ. 2006. How corticosteroids control inflammation: Quintiles Prize Lecture 2005. Br J Pharmacol 148(3):245–254. 16604091 10.1038/sj.bjp.0706736 PMC1751559

[B10] Bautista E, Arcos M, Jimenez-Alvarez L, Garcia-Sancho MC, Vazquez ME, Pena E, Higuera A, Ramirez G, Fernandez-Plata R, Cruz-Lagunas A, Garcia-Moreno SA, Urrea F, Ramirez R, Correa-Rotter R, Perez-Padilla JR, Zuniga J. 2013. Angiogenic and inflammatory markers in acute respiratory distress syndrome and renal injury associated to A/H1N1 virus infection. Exp Mol Pathol 94(3):486–492. 23542734 10.1016/j.yexmp.2013.03.007

[B11] Bayati A, Kumar R, Francis V, McPherson PS. 2021. SARS-CoV-2 infects cells after viral entry via clathrin-mediated endocytosis. J Biol Chem 296:100306. 33476648 10.1016/j.jbc.2021.100306 PMC7816624

[B12] Benson MJ, Dillon SR, Castigli E, Geha RS, Xu S, Lam KP, Noelle RJ. 2008. Cutting edge: the dependence of plasma cells and independence of memory B cells on BAFF and APRIL. J Immunol 180(6):3655–3659. 18322170 10.4049/jimmunol.180.6.3655

[B13] Bickel M. 1993. The role of interleukin-8 in inflammation and mechanisms of regulation. J Periodontol 64(5 Suppl):456–460. 8315568

[B14] Blyth GA, Chan WF, Webster RG, Magor KE. 2016. Duck interferon-inducible transmembrane protein 3 mediates restriction of influenza viruses. J Virol 90(1):103–116. 26468537 10.1128/JVI.01593-15 PMC4702568

[B15] Boomer JS, To K, Chang KC, Takasu O, Osborne DF, Walton AH, Bricker TL, Jarman SD, 2nd, Kreisel D, Krupnick AS, Srivastava A, Swanson PE, Green JM, Hotchkiss RS. 2011. Immunosuppression in patients who die of sepsis and multiple organ failure. JAMA 306(23):2594–2605. 22187279 10.1001/jama.2011.1829 PMC3361243

[B16] Bortolotti D, Gentili V, Rizzo S, Schiuma G, Beltrami S, Strazzabosco G, Fernandez M, Caccuri F, Caruso A, Rizzo R. 2021. TLR3 and TLR7 RNA sensor activation during SARS-CoV-2 infection. Microorganisms 9(9):1820. 34576716 10.3390/microorganisms9091820 PMC8465566

[B17] Bose M, Mitra B, Mukherjee P. 2021. Mucin signature as a potential tool to predict susceptibility to COVID-19. Physiol Rep 9(1):e14701. 33373502 10.14814/phy2.14701 PMC7771898

[B18] Brass AL, Huang IC, Benita Y, John SP, Krishnan MN, Feeley EM, Ryan BJ, Weyer JL, van der Weyden L, Fikrig E, Adams DJ, Xavier RJ, Farzan M, Elledge SJ. 2009. The IFITM proteins mediate cellular resistance to influenza A H1N1 virus, West Nile virus, and dengue virus. Cell 139(7):1243–1254. 20064371 10.1016/j.cell.2009.12.017 PMC2824905

[B19] Brincks EL, Katewa A, Kucaba TA, Griffith TS, Legge KL. 2008. CD8 T cells utilize TRAIL to control influenza virus infection. J Immunol 181(7):4918–4925. 18802095 10.4049/jimmunol.181.7.4918 PMC2610351

[B20] Brown DM, Dilzer AM, Meents DL, Swain SL. 2006. CD4 T cell-mediated protection from lethal influenza: perforin and antibody-mediated mechanisms give a one-two punch. J Immunol 177(5):2888–2898. 16920924 10.4049/jimmunol.177.5.2888

[B21] Buyse S, Teixeira L, Galicier L, Mariotte E, Lemiale V, Seguin A, Bertheau P, Canet E, de Labarthe A, Darmon M, Rybojad M, Schlemmer B, Azoulay E. 2010. Critical care management of patients with hemophagocytic lymphohistiocytosis. Intensive Care Med 36(10):1695–1702. 20532477 10.1007/s00134-010-1936-z

[B22] Camicia G, Pozner R, de Larrañaga G. 2014. Neutrophil extracellular traps in sepsis. Shock 42(4):286–294. 25004062 10.1097/SHK.0000000000000221

[B23] Camp JV, Jonsson CB. 2017. A role for neutrophils in viral respiratory disease. Front Immunol 8:550–550. 28553293 10.3389/fimmu.2017.00550 PMC5427094

[B24] Canna SW, Behrens EM. 2012. Making sense of the cytokine storm: a conceptual framework for understanding, diagnosing, and treating hemophagocytic syndromes. Pediatr Clin North Am 59(2):329–344. 22560573 10.1016/j.pcl.2012.03.002 PMC3368378

[B25] Caroppo F, Biolo G, Belloni Fortina A. 2020. SARS-CoV-2 asymptomatic infection in a patient under treatment with dupilumab. J Eur Acad Dermatol Venereol 34(8):e368. 10.1111/jdv.16619 PMC7272929 32386431

[B26] Carugno A, Raponi F, Locatelli AG, Vezzoli P, Gambini DM, Di Mercurio M, Robustelli Test E, Sena P. 2020. No evidence of increased risk for Coronavirus Disease 2019 (COVID-19) in patients treated with Dupilumab for atopic dermatitis in a high-epidemic area - Bergamo, Lombardy, Italy. J Eur Acad Dermatol Venereol 34(9):e433-e434. 32339362 10.1111/jdv.16552 PMC7267230

[B27] Casalino E, Antoniol S, Fidouh N, Choquet C, Lucet JC, Duval X, Visseaux B, Pereira L. 2017. Influenza virus infections among patients attending emergency department according to main reason to presenting to ED: a 3-year prospective observational study during seasonal epidemic periods. PLoS One 12(8):e0182191. 28813449 10.1371/journal.pone.0182191 PMC5558947

[B28] Cavaillon JM, Munoz C, Fitting C, Misset B, Carlet J. 1992. Circulating cytokines: the tip of the iceberg? Circ Shock 38(2):145–152. 1423923

[B29] Centers for Disease Control and Prevention. 2009. Swine influenza A (H1N1) infection in two children—Southern California, March-April 2009. MMWR Morb Mortal Wkly Rep 58(15):400–402. 19390508

[B30] Chen G, Wu D, Guo W, Cao Y, Huang D, Wang H, Wang T, Zhang X, Chen H, Yu H, Zhang X, Zhang M, Wu S, Song J, Chen T, Han M, Li S, Luo X, Zhao J, Ning Q. 2020. Clinical and immunological features of severe and moderate coronavirus disease 2019. J Clin Invest 130(5):2620–2629. 32217835 10.1172/JCI137244 PMC7190990

[B31] Chicheportiche Y, Chicheportiche R, Sizing I, Thompson J, Benjamin CB, Ambrose C, Dayer JM. 2002. Proinflammatory activity of TWEAK on human dermal fibroblasts and synoviocytes: blocking and enhancing effects of anti-TWEAK monoclonal antibodies. Arthritis Res 4(2):126–133. 11879548 10.1186/ar388 PMC83846

[B32] Choreño-Parra JA, Jiménez-Álvarez LA, Cruz-Lagunas A, Rodríguez-Reyna TS, Ramírez-Martínez G, Sandoval-Vega M, Hernández-García DL, Choreño-Parra EM, Balderas-Martínez YI, Martinez-Sánchez ME, Márquez-García E, Sciutto E, Moreno-Rodríguez J, Barreto-Rodríguez JO, Vázquez-Rojas H, Centeno-Sáenz GI, Alvarado-Peña N, Salinas-Lara C, Sánchez-Garibay C, Galeana-Cadena D, Hernández G, Mendoza-Milla C, Domínguez A, Granados J, Mena-Hernández L, Pérez-Buenfil L, Domínguez-Cheritt G, Cabello-Gutiérrez C, Luna-Rivero C, Salas-Hernández J, Santillán-Doherty P, Regalado J, Hernández-Martínez A, Orozco L, Ávila-Moreno F, García-Latorre EA, Hernández-Cárdenas CM, Khader SA, Zlotnik A, Zúñiga J. 2021a. Clinical and Immunological Factors That Distinguish COVID-19 From Pandemic Influenza A(H1N1). Front Immunol 12:593595. 33995342 10.3389/fimmu.2021.593595 PMC8115405

[B33] Choreño-Parra JA, Jiménez-Álvarez LA, Ramírez-Martínez G, Cruz-Lagunas A, Thapa M, Fernández-López LA, Carnalla-Cortés M, Choreño-Parra EM, Mena-Hernández L, Sandoval-Vega M, Hernández-Montiel EM, Hernández-García DL, Ramírez-Noyola JA, Reyes-López CE, Domínguez-Faure A, Zamudio-López GY, Márquez-García E, Moncada-Morales A, Mendoza-Milla C, Cervántes-Rosete D, Muñoz-Torrico M, Luna-Rivero C, García-Latorre EA, Guadarrama-Ortíz P, Ávila-Moreno F, Domínguez-Cherit G, Rodríguez-Reyna TS, Mudd PA, Hernández-Cárdenas CM, Khader SA, Zúñiga J. 2021b. Expression of Surfactant protein D (SP-D) distinguishes severe pandemic influenza A(H1N1) from COVID-19. J Infect Dis 224(1):21–30. 33668070 10.1093/infdis/jiab113 PMC7989215

[B34] Choreño-Parra JA, Jiménez-Álvarez LA, Ramírez-Martínez G, Sandoval-Vega M, Salinas-Lara C, Sánchez-Garibay C, Luna-Rivero C, Hernández-Montiel EM, Fernández-López LA, Cabrera-Cornejo MF, Choreño-Parra EM, Cruz-Lagunas A, Domínguez A, Márquez-García E, Cabello-Gutiérrez C, Bolaños-Morales FV, Mena-Hernández L, Delgado-Zaldivar D, Rebolledo-García D, Guadarrama-Ortiz P, Regino-Zamarripa NE, Mendoza-Milla C, García-Latorre EA, Rodiguez-Reyna TS, Cervántes-Rosete D, Hernández-Cárdenas CM, Khader SA, Zlotnik A, Zúñiga J. 2021c. CXCL17 is a specific diagnostic biomarker for severe pandemic influenza A(H1N1) that predicts poor clinical outcome. Front Immunol 12:633297. 33717172 10.3389/fimmu.2021.633297 PMC7953906

[B35] Choreno-Parra JA, Thirunavukkarasu S, Zuniga J, Khader SA. 2020. The protective and pathogenic roles of CXCL17 in human health and disease: potential in respiratory medicine. Cytokine Growth Factor Rev 53:53–62. 32345516 10.1016/j.cytogfr.2020.04.004 PMC7177079

[B36] Cohen J. 2002. The immunopathogenesis of sepsis. Nature 420(6917):885–891. 12490963 10.1038/nature01326

[B37] Collaborators GBDI. 2019. Mortality, morbidity, and hospitalisations due to influenza lower respiratory tract infections, 2017: an analysis for the Global Burden of Disease Study 2017. Lancet Respir Med 7(1):69–89. 30553848 10.1016/S2213-2600(18)30496-X PMC6302221

[B38] Costagliola G, Spada E, Consolini R. 2021. Age-related differences in the immune response could contribute to determine the spectrum of severity of COVID-19. Immun Inflamm Dis 9(2):331–339. 33566457 10.1002/iid3.404 PMC8014746

[B39] Cubuk J, Alston JJ, Incicco JJ, Singh S, Stuchell-Brereton MD, Ward MD, Zimmerman MI, Vithani N, Griffith D, Wagoner JA, Bowman GR, Hall KB, Soranno A, Holehouse AS. 2021. The SARS-CoV-2 nucleocapsid protein is dynamic, disordered, and phase separates with RNA. Nat Commun 12(1):1936. 33782395 10.1038/s41467-021-21953-3 PMC8007728

[B40] D'Armiento J, Dalal SS, Okada Y, Berg RA, Chada K. 1992. Collagenase expression in the lungs of transgenic mice causes pulmonary emphysema. Cell 71(6):955–961. 1458541 10.1016/0092-8674(92)90391-o

[B41] Dahlen B, Shute J, Howarth P. 1999. Immunohistochemical localisation of the matrix metalloproteinases MMP-3 and MMP-9 within the airways in asthma. Thorax 54(7):590–596. 10377203 10.1136/thx.54.7.590 PMC1745517

[B42] Dalskov L, Møhlenberg M, Thyrsted J, Blay-Cadanet J, Poulsen ET, Folkersen BH, Skaarup SH, Olagnier D, Reinert L, Enghild JJ, Hoffmann HJ, Holm CK, Hartmann R. 2020. SARS-CoV-2 evades immune detection in alveolar macrophages. EMBO Rep 21(12):e51252. 33112036 10.15252/embr.202051252 PMC7645910

[B43] De Backer D, Donadello K, Taccone FS, Ospina-Tascon G, Salgado D, Vincent JL. 2011. Microcirculatory alterations: potential mechanisms and implications for therapy. Ann Intensive Care 1(1):27. 21906380 10.1186/2110-5820-1-27 PMC3224481

[B44] de Jong MD, Simmons CP, Thanh TT, Hien VM, Smith GJ, Chau TN, Hoang DM, Chau NV, Khanh TH, Dong VC, Qui PT, Cam BV, Ha do Q, Guan Y, Peiris JS, Chinh NT, Hien TT, Farrar J. 2006. Fatal outcome of human influenza A (H5N1) is associated with high viral load and hypercytokinemia. Nat Med 12(10):1203–1207. 16964257 10.1038/nm1477 PMC4333202

[B45] De Luca G, Cavalli G, Campochiaro C, Della-Torre E, Angelillo P, Tomelleri A, Boffini N, Tentori S, Mette F, Farina N, Rovere-Querini P, Ruggeri A, D'Aliberti T, Scarpellini P, Landoni G, De Cobelli F, Paolini JF, Zangrillo A, Tresoldi M, Trapnell BC, Ciceri F, Dagna L. 2020. GM-CSF blockade with mavrilimumab in severe COVID-19 pneumonia and systemic hyperinflammation: a single-centre, prospective cohort Study. Lancet Rheumatol 2(8):e465–e473. 32835256 10.1016/S2665-9913(20)30170-3 PMC7430344

[B46] Dunning J, Thwaites RS, Openshaw PJM. 2020. Seasonal and pandemic influenza: 100 years of progress, still much to learn. Mucosal Immunol 13(4):566–573. 32317736 10.1038/s41385-020-0287-5 PMC7223327

[B47] Eisenbarth SC, Flavell RA. 2009. Innate instruction of adaptive immunity revisited: the inflammasome. EMBO Mol Med 1(2):92–98. 20049709 10.1002/emmm.200900014 PMC3378119

[B48] Emmenegger U, Schaer DJ, Larroche C, Neftel KA. 2005. Haemophagocytic syndromes in adults: current concepts and challenges ahead. Swiss Med Wkly 135(21–22):299–314. 16034684 10.4414/smw.2005.10976

[B49] Engelmann B, Massberg S. 2013. Thrombosis as an intravascular effector of innate immunity. Nat Rev Immunol 13(1):34–45. 23222502 10.1038/nri3345

[B50] Estella A. 2011. Cytokine levels in bronchoalveolar lavage and serum in 3 patients with 2009 Influenza A(H1N1)v severe pneumonia. J Infect Dev Ctries 5(7):540–543. 21795823 10.3855/jidc.1618

[B51] Everitt AR, Clare S, Pertel T, John SP, Wash RS, Smith SE, Chin CR, Feeley EM, Sims JS, Adams DJ, Wise HM, Kane L, Goulding D, Digard P, Anttila V, Baillie JK, Walsh TS, Hume DA, Palotie A, Xue Y, Colonna V, Tyler-Smith C, Dunning J, Gordon SB, Everingham K, Dawson H, Hope D, Ramsay P, Walsh TS, Campbell A, Kerr S, Harrison D, Rowan K, Addison J, Donald N, Galt S, Noble D, Taylor J, Webster N, Taylor I, Aldridge J, Dornan R, Richard C, Gilmour D, Simmons R, White R, Jardine C, Williams D, Booth M, Quasim T, Watson V, Henry P, Munro F, Bell L, Ruddy J, Cole S, Southward J, Allcoat P, Gray S, McDougall M, Matheson J, Whiteside J, Alcorn D, Rooney K, Sundaram R, Imrie G, Bruce J, McGuigan K, Moultrie S, Cairns C, Grant J, Hughes M, Murdoch C, Davidson A, Harris G, Paterson R, Wallis C, Binning S, Pollock M, Antonelli J, Duncan A, Gibson J, McCulloch C, Murphy L, Haley C, Faulkner G, Freeman T, Hume DA, Baillie JK, Chaussabel D, Adamson WE, Carman WF, Thompson C, Zambon MC, Aylin P, Ashby D, Barclay WS, Brett SJ, Cookson WO, Drumright LN, Dunning J, Elderfield RA, Garcia-Alvarez L, Gazzard BG, Griffiths MJ, Habibi MS, Hansel TT, Herberg JA, Holmes AH, Hussell T, Johnston SL, Kon OM, Levin M, Moffatt MF, Nadel S, Openshaw PJ, Warner JO, Aston SJ, Gordon SB, Hay A, McCauley J, O'Garra A, Banchereau J, Hayward A, Kellam P, Baillie JK, Hume DA, Simmonds P, McNamara PS, Semple MG, Smyth RL, Nguyen-Van-Tam JS, Ho LP, McMichael AJ, Kellam P, Smyth RL, Openshaw PJ, Dougan G, Brass AL, Kellam P, The Gen II, Critical Care Medicine UoE, Generation Scotland UoEMMC, Intensive Care National A, Research Centre L, Intensive Care Unit ARI, Intensive Care Unit AH, Intensive Care Unit BGHM, Intensive Care Unit CHK, Intensive Care Unit D, Galloway Royal I, Intensive Care Unit GRI, Intensive Care Unit HHL, Intensive Care Unit IRHG, Intensive Care Unit MHA, Intensive Care Unit NHD, Intensive Care Unit QMHD, Intensive Care Unit RHI, Intensive Care Unit RAHP, Intensive Care Unit SGHG, Intensive Care Unit SJsHL, Intensive Care Unit SRI, Intensive Care Unit SHG, Intensive Care Unit VHG, Intensive Care Unit WGHE, Intensive Care Unit WIG, Wellcome Trust Clinical Research Facility E, Roslin Institute UoE, The MI, Benaroya Research Institute USA, Gartnavel General Hospital GGUK, Health Protection Agency UK, Imperial College London UK, Liverpool School of Tropical Medicine UK, National Institute for Medical Research UK, Roche NUSA, University College London UK, University of Edinburgh UK, University of Liverpool UK, University of Nottingham UK, University of Oxford UK, Wellcome Trust Sanger Institute UK. 2012. IFITM3 restricts the morbidity and mortality associated with influenza. Nature 484(7395):519–523. 22446628 10.1038/nature10921 PMC3648786

[B52] Ewer KJ, Barrett JR, Belij-Rammerstorfer S, Sharpe H, Makinson R, Morter R, Flaxman A, Wright D, Bellamy D, Bittaye M, Dold C, Provine NM, Aboagye J, Fowler J, Silk SE, Alderson J, Aley PK, Angus B, Berrie E, Bibi S, Cicconi P, Clutterbuck EA, Chelysheva I, Folegatti PM, Fuskova M, Green CM, Jenkin D, Kerridge S, Lawrie A, Minassian AM, Moore M, Mujadidi Y, Plested E, Poulton I, Ramasamy MN, Robinson H, Song R, Snape MD, Tarrant R, Voysey M, Watson MEE, Douglas AD, Hill AVS, Gilbert SC, Pollard AJ, Lambe T, Ali A, Allen E, Baker M, Barnes E, Borthwick N, Boyd A, Brown-O'Sullivan C, Burgoyne J, Byard N, Puig IC, Cappuccini F, Cho J-S, Cicconi P, Clark E, Crocker WEM, Datoo MS, Davies H, Donnellan FR, Dunachie SJ, Edwards NJ, Elias SC, Furze J, Gilbride C, Gorini G, Gupta G, Harris SA, Hodgson SHC, Hou MM, Jackson S, Jones K, Kailath R, King L, Larkworthy CW, Li Y, Lias AM, Linder A, Lipworth S, Ramon RL, Madhavan M, Marlow E, Marshall JL, Mentzer AJ, Morrison H, Moya N, Mukhopadhyay E, Noé A, Nugent FL, Pipini D, Pulido-Gomez D, Lopez FR, Ritchie AJ, Rudiansyah I, Salvador S, Sanders H, Satti I, Shea A, Silk S, Spencer AJ, Tanner R, Taylor IJ, Themistocleous Y, Thomas M, Tran N, Truby A, Turner C, Turner N, Ulaszewska M, Worth AT, Kingham-Page L, Alvarez MPP, Anslow R, Bates L, Beadon K, Beckley R, Beveridge A, Bijker EM, Blackwell L, Burbage J, Camara S, Carr M, Colin-Jones R, Cooper R, Cunningham CJ, Demissie T, Maso CD, Douglas N, Drake-Brockman R, Drury RE, Emary KRW, Felle S, Feng S, Silva CFD, Ford KJ, Francis E, Gracie L, Hamlyn J, Hanumunthadu B, Harrison D, Hart TC, Hawkins S, Hill J, Howe E, Howell N, Jones E, Keen J, Kelly S, Kerr D, Khan L, Kinch J, Koleva S, Lees EA, Lelliott A, Liu X, Marchevsky NG, Marinou S, McEwan J, Morey E, Morshead G, Muller J, Munro C, Murphy S, Mweu P, Nuthall E, O'Brien K, O'Connor D, O'Reilly PJ, Oguti B, Osborne P, Owino N, Parker K, Pfafferott K, Phillips D, Provstgaard-Morys S, Ratcliffe H, Rawlinson T, Rhead S, Roberts H, Sanders K, Silva-Reyes L, Rollier CS, Smith CC, Smith DJ, Stockdale L, Szigeti A, Thomas TM, Thompson A, Tomic A, Tonks S, Varughese R, Verheul MK, Vichos I, Walker L, White C, White R, Yao XL, Conlon CP, Frater J, Cifuentes L, Baleanu I, Bolam E, Boland E, Brenner T, Damratoski BE, Datta C, Muhanna OE, Fisher R, Galian-Rubio P, Hodges G, Jackson F, Liu S, Loew L, Morgans R, Morris SJ, Olchawski V, Oliveria C, Parracho H, Pabon ER, Tahiri-Alaoui A, Taylor K, Williams P, Zizi D, Arbe-Barnes EH, Baker P, Batten A, Downing C, Drake J, English MR, Henry JA, Iveson P, Killen A, King TB, Larwood JPJ, Mallett G, Mansatta K, Mirtorabi N, Patrick-Smith M, Perring J, Radia K, Roche S, Schofield E, Naude RtW, Towner J, Baker N, Bewley KR, Brunt E, Buttigieg KR, Carroll MW, Charlton S, Coombes NS, Elmore MJ, Godwin K, Hallis B, Knott D, McInroy L, Shaik I, Thomas K, Tree JA, Blundell CL, Cao M, Kelly D, Schmid A, Skelly DT, Themistocleous A, Dong T, Field S, Hamilton E, Kelly E, Klenerman P, Knight JC, Lie Y, Petropoulos C, Sedik C, Wrin T, Meddaugh G, Peng Y, Screaton G, Stafford E, the Oxford CVTG. 2021. T cell and antibody responses induced by a single dose of ChAdOx1 nCoV-19 (AZD1222) vaccine in a phase 1/2 clinical trial. Nat Med 27(2):270–278. 33335323 10.1038/s41591-020-01194-5

[B53] Fajgenbaum DC, June CH. 2020. Cytokine storm. N Engl J Med 383(23):2255–2273. 33264547 10.1056/NEJMra2026131 PMC7727315

[B54] Ferrara JL, Abhyankar S, Gilliland DG. 1993. Cytokine storm of graft-versus-host disease: a critical effector role for interleukin-1. Transplant Proc 25(1 Pt 2):1216–1217. 8442093

[B55] Ferreira-Gomes M, Kruglov A, Durek P, Heinrich F, Tizian C, Heinz GA, Pascual-Reguant A, Du W, Mothes R, Fan C, Frischbutter S, Habenicht K, Budzinski L, Ninnemann J, Jani PK, Guerra GM, Lehmann K, Matz M, Ostendorf L, Heiberger L, Chang HD, Bauherr S, Maurer M, Schönrich G, Raftery M, Kallinich T, Mall MA, Angermair S, Treskatsch S, Dörner T, Corman VM, Diefenbach A, Volk HD, Elezkurtaj S, Winkler TH, Dong J, Hauser AE, Radbruch H, Witkowski M, Melchers F, Radbruch A, Mashreghi MF. 2021. SARS-CoV-2 in severe COVID-19 induces a TGF-β-dominated chronic immune response that does not target itself. Nat Commun 12(1):1961. 33785765 10.1038/s41467-021-22210-3 PMC8010106

[B56] Ferrucci S, Romagnuolo M, Angileri L, Berti E, Tavecchio S. 2020. Safety of dupilumab in severe atopic dermatitis and infection of Covid-19: two case reports. J Eur Acad Dermatol Venereol 34(7):e303-e304. 32330323 10.1111/jdv.16527 PMC7267596

[B57] Fiore-Gartland A, Panoskaltsis-Mortari A, Agan AA, Mistry AJ, Thomas PG, Matthay MA, Hertz T, Randolph AG. 2017. Cytokine profiles of severe influenza virus-related complications in children. Front Immunol 8:1423. 29163498 10.3389/fimmu.2017.01423 PMC5681736

[B58] Folegatti PM, Ewer KJ, Aley PK, Angus B, Becker S, Belij-Rammerstorfer S, Bellamy D, Bibi S, Bittaye M, Clutterbuck EA, Dold C, Faust SN, Finn A, Flaxman AL, Hallis B, Heath P, Jenkin D, Lazarus R, Makinson R, Minassian AM, Pollock KM, Ramasamy M, Robinson H, Snape M, Tarrant R, Voysey M, Green C, Douglas AD, Hill AVS, Lambe T, Gilbert SC, Pollard AJ, Aboagye J, Adams K, Ali A, Allen E, Allison JL, Anslow R, Arbe-Barnes EH, Babbage G, Baillie K, Baker M, Baker N, Baker P, Baleanu I, Ballaminut J, Barnes E, Barrett J, Bates L, Batten A, Beadon K, Beckley R, Berrie E, Berry L, Beveridge A, Bewley KR, Bijker EM, Bingham T, Blackwell L, Blundell CL, Bolam E, Boland E, Borthwick N, Bower T, Boyd A, Brenner T, Bright PD, Brown-O'Sullivan C, Brunt E, Burbage J, Burge S, Buttigieg KR, Byard N, Cabera Puig I, Calvert A, Camara S, Cao M, Cappuccini F, Carr M, Carroll MW, Carter V, Cathie K, Challis RJ, Charlton S, Chelysheva I, Cho J-S, Cicconi P, Cifuentes L, Clark H, Clark E, Cole T, Colin-Jones R, Conlon CP, Cook A, Coombes NS, Cooper R, Cosgrove CA, Coy K, Crocker WEM, Cunningham CJ, Damratoski BE, Dando L, Datoo MS, Davies H, De Graaf H, Demissie T, Di Maso C, Dietrich I, Dong T, Donnellan FR, Douglas N, Downing C, Drake J, Drake-Brockman R, Drury RE, Dunachie SJ, Edwards NJ, Edwards FDL, Edwards CJ, Elias SC, Elmore MJ, Emary KRW, English MR, Fagerbrink S, Felle S, Feng S, Field S, Fixmer C, Fletcher C, Ford KJ, Fowler J, Fox P, Francis E, Frater J, Furze J, Fuskova M, Galiza E, Gbesemete D, Gilbride C, Godwin K, Gorini G, Goulston L, Grabau C, Gracie L, Gray Z, Guthrie LB, Hackett M, Halwe S, Hamilton E, Hamlyn J, Hanumunthadu B, Harding I, Harris SA, Harris A, Harrison D, Harrison C, Hart TC, Haskell L, Hawkins S, Head I, Henry JA, Hill J, Hodgson SHC, Hou MM, Howe E, Howell N, Hutlin C, Ikram S, Isitt C, Iveson P, Jackson S, Jackson F, James SW, Jenkins M, Jones E, Jones K, Jones CE, Jones B, Kailath R, Karampatsas K, Keen J, Kelly S, Kelly D, Kerr D, Kerridge S, Khan L, Khan U, Killen A, Kinch J, King TB, King L, King J, Kingham-Page L, Klenerman P, Knapper F, Knight JC, Knott D, Koleva S, Kupke A, Larkworthy CW, Larwood JPJ, Laskey A, Lawrie AM, Lee A, Ngan Lee KY, Lees EA, Legge H, Lelliott A, Lemm N-M, Lias AM, Linder A, Lipworth S, Liu X, Liu S, Lopez Ramon R, Lwin M, Mabesa F, Madhavan M, Mallett G, Mansatta K, Marcal I, Marinou S, Marlow E, Marshall JL, Martin J, McEwan J, McInroy L, Meddaugh G, Mentzer AJ, Mirtorabi N, Moore M, Moran E, Morey E, Morgan V, Morris SJ, Morrison H, Morshead G, Morter R, Mujadidi YF, Muller J, Munera-Huertas T, Munro C, Munro A, Murphy S, Munster VJ, Mweu P, Noé A, Nugent FL, Nuthall E, O'Brien K, O'Connor D, Oguti B, Oliver JL, Oliveira C, O'Reilly PJ, Osborn M, Osborne P, Owen C, Owens D, Owino N, Pacurar M, Parker K, Parracho H, Patrick-Smith M, Payne V, Pearce J, Peng Y, Peralta Alvarez MP, Perring J, Pfafferott K, Pipini D, Plested E, Pluess-Hall H, Pollock K, Poulton I, Presland L, Provstgaard-Morys S, Pulido D, Radia K, Ramos Lopez F, Rand J, Ratcliffe H, Rawlinson T, Rhead S, Riddell A, Ritchie AJ, Roberts H, Robson J, Roche S, Rohde C, Rollier CS, Romani R, Rudiansyah I, Saich S, Sajjad S, Salvador S, Sanchez Riera L, Sanders H, Sanders K, Sapaun S, Sayce C, Schofield E, Screaton G, Selby B, Semple C, Sharpe HR, Shaik I, Shea A, Shelton H, Silk S, Silva-Reyes L, Skelly DT, Smee H, Smith CC, Smith DJ, Song R, Spencer AJ, Stafford E, Steele A, Stefanova E, Stockdale L, Szigeti A, Tahiri-Alaoui A, Tait M, Talbot H, Tanner R, Taylor IJ, Taylor V, Te Water Naude R, Thakur N, Themistocleous Y, Themistocleous A, Thomas M, Thomas TM, Thompson A, Thomson-Hill S, Tomlins J, Tonks S, Towner J, Tran N, Tree JA, Truby A, Turkentine K, Turner C, Turner N, Turner S, Tuthill T, Ulaszewska M, Varughese R, Van Doremalen N, Veighey K, Verheul MK, Vichos I, Vitale E, Walker L, Watson MEE, Welham B, Wheat J, White C, White R, Worth AT, Wright D, Wright S, Yao XL, Yau Y. 2020. Safety and immunogenicity of the ChAdOx1 nCoV-19 vaccine against SARS-CoV-2: a preliminary report of a phase 1/2, single-blind, randomised controlled trial. Lancet 396(10249):467–478. 32702298 10.1016/S0140-6736(20)31604-4 PMC7445431

[B59] Forbester JL, Humphreys IR. 2021. Genetic influences on viral-induced cytokine responses in the lung. Mucosal Immunol 14(1):14–25. 33184476 10.1038/s41385-020-00355-6 PMC7658619

[B60] Fraissé M, Logre E, Mentec H, Cally R, Plantefève G, Contou D. 2020. Eosinophilia in critically ill COVID-19 patients: a French monocenter retrospective study. Critic Care 24(1):635. 10.1186/s13054-020-03361-z PMC7607895 33143729

[B61] Fukaya S, Yasuda S, Hashimoto T, Oku K, Kataoka H, Horita T, Atsumi T, Koike T. 2008. Clinical features of haemophagocytic syndrome in patients with systemic autoimmune diseases: analysis of 30 cases. Rheumatology (Oxford) 47(11):1686–1691. 18782855 10.1093/rheumatology/ken342

[B62] Gabrilovich DI, Nagaraj S. 2009. Myeloid-derived suppressor cells as regulators of the immune system. Nat Rev Immunol 9(3):162–174. 19197294 10.1038/nri2506 PMC2828349

[B63] Galley HF. 2011. Oxidative stress and mitochondrial dysfunction in sepsis. Br J Anaesth 107(1):57–64. 21596843 10.1093/bja/aer093

[B64] Gao R, Bhatnagar J, Blau DM, Greer P, Rollin DC, Denison AM, Deleon-Carnes M, Shieh W-J, Sambhara S, Tumpey TM, Patel M, Liu L, Paddock C, Drew C, Shu Y, Katz JM, Zaki SR. 2013. Cytokine and chemokine profiles in lung tissues from fatal cases of 2009 pandemic influenza A (H1N1): role of the host immune response in pathogenesis. Am J Pathol 183(4):1258–1268. 23938324 10.1016/j.ajpath.2013.06.023 PMC7119452

[B65] Ghebrehewet S, MacPherson P, Ho A. 2016. Influenza. BMJ 355:i6258. 27927672 10.1136/bmj.i6258 PMC5141587

[B66] Glowacka I, Bertram S, Muller MA, Allen P, Soilleux E, Pfefferle S, Steffen I, Tsegaye TS, He Y, Gnirss K, Niemeyer D, Schneider H, Drosten C, Pohlmann S. 2011. Evidence that TMPRSS2 activates the severe acute respiratory syndrome coronavirus spike protein for membrane fusion and reduces viral control by the humoral immune response. J Virol 85(9):4122–4134. 21325420 10.1128/JVI.02232-10 PMC3126222

[B67] Gogos CA, Drosou E, Bassaris HP, Skoutelis A. 2000. Pro- versus Anti-inflammatory cytokine profile in patients with severe sepsis: a marker for prognosis and future therapeutic options. J Infect Dis 181(1):176–180. 10608764 10.1086/315214

[B68] Goldenberg NM, Steinberg BE, Slutsky AS, Lee WL. 2011. Broken barriers: a new take on sepsis pathogenesis. Sci Transl Med 3(88):88ps25. 10.1126/scitranslmed.3002011 21697528

[B69] Gómez J, Albaiceta GM, Cuesta-Llavona E, García-Clemente M, López-Larrea C, Amado-Rodríguez L, López-Alonso I, Melón S, Alvarez-Argüelles ME, Gil-Peña H, Vidal-Castiñeira JR, Corte-Iglesias V, Saiz ML, Alvarez V, Coto E. 2021. The Interferon-induced transmembrane protein 3 gene (IFITM3) rs12252 C variant is associated with COVID-19. Cytokine 137:155354. 33113474 10.1016/j.cyto.2020.155354

[B70] Gong F, Pan YH, Huang X, Zhu HY, Jiang DL. 2017. From bench to bedside: therapeutic potential of interleukin-9 in the treatment of asthma (Review). Exp Ther Med 13(2):389–394. 28352305 10.3892/etm.2017.4024 PMC5347659

[B71] Greco E, Lupia E, Bosco O, Vizio B, Montrucchio G. 2017. Platelets and multi-organ failure in sepsis. Int J Mol Sci 18(10):2200. 10.3390/ijms18102200 PMC5666881 29053592

[B72] Greenlee KJ, Werb Z, Kheradmand F. 2007. Matrix metalloproteinases in lung: multiple, multifarious, and multifaceted. Physiol Rev 87(1):69–98. 17237343 10.1152/physrev.00022.2006 PMC2656382

[B73] Groom JR, Luster AD. 2011. CXCR3 ligands: redundant, collaborative and antagonistic functions. Immunol Cell Biol 89(2):207–215. 21221121 10.1038/icb.2010.158 PMC3863330

[B74] Group RC, Horby P, Lim WS, Emberson JR, Mafham M, Bell JL, Linsell L, Staplin N, Brightling C, Ustianowski A, Elmahi E, Prudon B, Green C, Felton T, Chadwick D, Rege K, Fegan C, Chappell LC, Faust SN, Jaki T, Jeffery K, Montgomery A, Rowan K, Juszczak E, Baillie JK, Haynes R, Landray MJ. 2021. Dexamethasone in hospitalized patients with Covid-19. N Engl J Med 384(8):693–704. 32678530 10.1056/NEJMoa2021436 PMC7383595

[B75] Guo J, Wang S, Xia H, Shi D, Chen Y, Zheng S, Chen Y, Gao H, Guo F, Ji Z, Huang C, Luo R, Zhang Y, Zuo J, Chen Y, Xu Y, Xia J, Zhu C, Xu X, Qiu Y, Sheng J, Xu K, Li L. 2021. Cytokine signature associated with disease severity in COVID-19. Front Immunol 12:681516. 34489933 10.3389/fimmu.2021.681516 PMC8418386

[B76] Hadjadj J, Yatim N, Barnabei L, Corneau A, Boussier J, Smith N, Pere H, Charbit B, Bondet V, Chenevier-Gobeaux C, Breillat P, Carlier N, Gauzit R, Morbieu C, Pene F, Marin N, Roche N, Szwebel TA, Merkling SH, Treluyer JM, Veyer D, Mouthon L, Blanc C, Tharaux PL, Rozenberg F, Fischer A, Duffy D, Rieux-Laucat F, Kerneis S, Terrier B. 2020. Impaired type I interferon activity and inflammatory responses in severe COVID-19 patients. Science 369(6504):718–724. 32661059 10.1126/science.abc6027 PMC7402632

[B77] Hamming I, Timens W, Bulthuis ML, Lely AT, Navis G, van Goor H. 2004. Tissue distribution of ACE2 protein, the functional receptor for SARS coronavirus. A first step in understanding SARS pathogenesis. J Pathol 203(2):631–637. 15141377 10.1002/path.1570 PMC7167720

[B78] Han H, Ma Q, Li C, Liu R, Zhao L, Wang W, Zhang P, Liu X, Gao G, Liu F, Jiang Y, Cheng X, Zhu C, Xia Y. 2020. Profiling serum cytokines in COVID-19 patients reveals IL-6 and IL-10 are disease severity predictors. Emerg Microbes Infect 9(1):1123–1130. 32475230 10.1080/22221751.2020.1770129 PMC7473317

[B79] Han S, Mallampalli RK. 2015. The role of surfactant in lung disease and host defense against pulmonary infections. Ann Am Thorac Soc 12(5):765–774. 25742123 10.1513/AnnalsATS.201411-507FR PMC4418337

[B80] Hansson GC. 2019. Mucus and mucins in diseases of the intestinal and respiratory tracts. J Intern Med 285(5):479–490. 30963635 10.1111/joim.12910 PMC6497544

[B81] Hernández-Cárdenas CM, Choreño-Parra JA, Torruco-Sotelo C, Jurado F, Serna-Secundino H, Aguilar C, García-Olazarán JG, Hernández-García D, Choreño-Parra EM, Zúñiga J, Lugo-Goytia G. 2021. Clinical Risk Factors for Mortality Among Critically Ill Mexican Patients With COVID-19. Front Med (Lausanne) 8:699607. 34513872 10.3389/fmed.2021.699607 PMC8429783

[B82] Herold S, Becker C, Ridge KM, Budinger GR. 2015. Influenza virus-induced lung injury: pathogenesis and implications for treatment. Eur Respir J 45(5):1463–1478. 25792631 10.1183/09031936.00186214

[B83] Hoffmann M, Kleine-Weber H, Schroeder S, Kruger N, Herrler T, Erichsen S, Schiergens TS, Herrler G, Wu NH, Nitsche A, Muller MA, Drosten C, Pohlmann S. 2020. SARS-CoV-2 cell entry depends on ACE2 and TMPRSS2 and is blocked by a clinically proven protease inhibitor. Cell 181(2):271–280 e8. 32142651 10.1016/j.cell.2020.02.052 PMC7102627

[B84] Holly JMP, Biernacka K, Maskell N, Perks CM. 2020. Obesity, Diabetes and COVID-19: an infectious disease spreading from the east collides with the consequences of an unhealthy western lifestyle. Front Endocrinol 11:582870. 10.3389/fendo.2020.582870 PMC7527410 33042029

[B85] Honce R, Schultz-Cherry S. 2019. Impact of obesity on influenza a virus pathogenesis, immune response, and evolution. Front Immunol 10:1071. 31134099 10.3389/fimmu.2019.01071 PMC6523028

[B86] Hotchkiss RS, Tinsley KW, Swanson PE, Grayson MH, Osborne DF, Wagner TH, Cobb JP, Coopersmith C, Karl IE. 2002. Depletion of dendritic cells, but not macrophages, in patients with sepsis. J Immunol 168(5):2493–2500. 11859143 10.4049/jimmunol.168.5.2493

[B87] Hotchkiss RS, Tinsley KW, Swanson PE, Schmieg RE Jr., Hui JJ, Chang KC, Osborne DF, Freeman BD, Cobb JP, Buchman TG, Karl IE. 2001. Sepsis-induced apoptosis causes progressive profound depletion of B and CD4+ T lymphocytes in humans. J Immunol 166(11):6952–6963. 11359857 10.4049/jimmunol.166.11.6952

[B88] Hsieh M-H, Beirag N, Murugaiah V, Chou Y-C, Kuo W-S, Kao H-F, Madan T, Kishore U, Wang J-Y. 2021. Human surfactant protein D binds spike protein and acts as an entry inhibitor of SARS-CoV-2 pseudotyped viral particles. Front Immunol 12(1613):641360. 34054808 10.3389/fimmu.2021.641360 PMC8161545

[B89] Huang C WY, Li X, Ren L, Zhao J, Hu Y, Zhang L, Fan G, Xu J, Gu X, Cheng, Z YT, Xia J, Wei Y, Wu W, Xie X, Yin W, Li H, Liu M, Xiao Y, Gao H, Guo L, Xie J WG, Jiang R, Gao Z, Jin Q, Wang J, Cao B. 2020. Clinical features of patients infected with 2019 novel coronavirus in Wuhan, China. Lancet 395(10223):497–506. 31986264 10.1016/S0140-6736(20)30183-5 PMC7159299

[B90] Iba T, Levi M, Levy JH. 2020. Sepsis-induced coagulopathy and disseminated intravascular coagulation. Semin Thromb Hemost 46(1):89–95. 31443111 10.1055/s-0039-1694995

[B91] Iba T, Levy JH. 2018. Inflammation and thrombosis: roles of neutrophils, platelets and endothelial cells and their interactions in thrombus formation during sepsis. J Thromb Haemost 16(2):231–241. 29193703 10.1111/jth.13911

[B92] Ichikawa A, Kuba K, Morita M, Chida S, Tezuka H, Hara H, Sasaki T, Ohteki T, Ranieri VM, dos Santos CC, Kawaoka Y, Akira S, Luster AD, Lu B, Penninger JM, Uhlig S, Slutsky AS, Imai Y. 2013. CXCL10-CXCR3 enhances the development of neutrophil-mediated fulminant lung injury of viral and nonviral origin. Am J Respir Crit Care Med 187(1):65–77. 23144331 10.1164/rccm.201203-0508OC PMC3927876

[B93] Ichinohe T, Pang IK, Iwasaki A. 2010. Influenza virus activates inflammasomes via its intracellular M2 ion channel. Nat Immunol 11(5):404–410. 20383149 10.1038/ni.1861 PMC2857582

[B94] Ito T, Liu YJ, Arima K. 2012. Cellular and molecular mechanisms of TSLP function in human allergic disorders—TSLP programs the “Th2 code” in dendritic cells. Allergol Int 61(1):35–43. 22189594 10.2332/allergolint.11-RAI-0376 PMC3660852

[B95] Ito Y, Abril ER, Bethea NW, McCuskey MK, Cover C, Jaeschke H, McCuskey RS. 2006. Mechanisms and pathophysiological implications of sinusoidal endothelial cell gap formation following treatment with galactosamine/endotoxin in mice. Am J Physiol Gastrointest Liver Physiol 291(2):G211–G218. 16574994 10.1152/ajpgi.00312.2005

[B96] Iwasaki A, Medzhitov R. 2004. Toll-like receptor control of the adaptive immune responses. Nat Immunol 5(10):987–995. 15454922 10.1038/ni1112

[B97] Jegaskanda S, Vanderven HA, Tan HX, Alcantara S, Wragg KM, Parsons MS, Chung AW, Juno JA, Kent SJ. 2019. Influenza virus infection enhances antibody-mediated NK cell functions via type I interferon-dependent pathways. J Virol 93(5):e02090-18. 30541850 10.1128/JVI.02090-18 PMC6384076

[B98] Jia R, Pan Q, Ding S, Rong L, Liu SL, Geng Y, Qiao W, Liang C. 2012. The N-terminal region of IFITM3 modulates its antiviral activity by regulating IFITM3 cellular localization. J Virol 86(24):13697–13707. 23055554 10.1128/JVI.01828-12 PMC3503121

[B99] Jiang C, Yao X, Zhao Y, Wu J, Huang P, Pan C, Liu S, Pan C. 2020. Comparative review of respiratory diseases caused by coronaviruses and influenza A viruses during epidemic season. Microbes Infect 22(6–7):236–244. 32405236 10.1016/j.micinf.2020.05.005 PMC7217786

[B100] Jørgensen SE, Christiansen M, Ryø LB, Gad HH, Gjedsted J, Staeheli P, Mikkelsen JG, Storgaard M, Hartmann R, Mogensen TH. 2018. Defective RNA sensing by RIG-I in severe influenza virus infection. Clin Exp Immunol 192(3):366–376. 29453856 10.1111/cei.13120 PMC5980616

[B101] Kaneko N, Kuo HH, Boucau J, Farmer JR, Allard-Chamard H, Mahajan VS, Piechocka-Trocha A, Lefteri K, Osborn M, Bals J, Bartsch YC, Bonheur N, Caradonna TM, Chevalier J, Chowdhury F, Diefenbach TJ, Einkauf K, Fallon J, Feldman J, Finn KK, Garcia-Broncano P, Hartana CA, Hauser BM, Jiang C, Kaplonek P, Karpell M, Koscher EC, Lian X, Liu H, Liu J, Ly NL, Michell AR, Rassadkina Y, Seiger K, Sessa L, Shin S, Singh N, Sun W, Sun X, Ticheli HJ, Waring MT, Zhu AL, Alter G, Li JZ, Lingwood D, Schmidt AG, Lichterfeld M, Walker BD, Yu XG, Padera RF Jr., Pillai S. 2020. Loss of Bcl-6-expressing T follicular helper cells and germinal centers in COVID-19. Cell 183(1):143–157.e13. 32877699 10.1016/j.cell.2020.08.025 PMC7437499

[B102] Kawada J, Kitagawa Y, Iwata N, Ito Y. 2013. Clinical characteristics of influenza virus infection in juvenile idiopathic arthritis patients treated with tocilizumab. Mod Rheumatol 23(5):972–976. 23070362 10.1007/s10165-012-0780-0

[B103] Kim Y-C, Jeong M-J, Jeong B-H. 2020. Strong association of regulatory single nucleotide polymorphisms (SNPs) of the IFITM3 gene with influenza H1N1 2009 pandemic virus infection. Cell Mol Immunol 17(6):662–664. 31685927 10.1038/s41423-019-0322-1 PMC7264285

[B104] Kim YC, Won SY, Jeong BH. 2021. The first association study of single-nucleotide polymorphisms (SNPs) of the IFITM1 gene with influenza H1N1 2009 pandemic virus infection. Mol Cell Toxicol 17(2):179–186. 33613683 10.1007/s13273-021-00123-y PMC7883877

[B105] Klein SL, Hodgson A, Robinson DP. 2012. Mechanisms of sex disparities in influenza pathogenesis. J Leukoc Biol 92(1):67–73. 22131346 10.1189/jlb.0811427 PMC4046247

[B106] Koch S, Sopel N, Finotto S. 2017. Th9 and other IL-9-producing cells in allergic asthma. Semin Immunopathol 39(1):55–68. 27858144 10.1007/s00281-016-0601-1

[B107] Kong Y, Han J, Wu X, Zeng H, Liu J, Zhang H. 2020. VEGF-D: a novel biomarker for detection of COVID-19 progression. Critic Care 24(1):373. 10.1186/s13054-020-03079-y PMC7309201 32576222

[B108] Korn T, Bettelli E, Oukka M, Kuchroo VK. 2009. IL-17 and Th17 Cells. Annu Rev Immunol 27:485–517. 19132915 10.1146/annurev.immunol.021908.132710

[B109] Kotch C, Barrett D, Teachey DT. 2019. Tocilizumab for the treatment of chimeric antigen receptor T cell-induced cytokine release syndrome. Expert Rev Clin Immunol 15(8):813–822. 31219357 10.1080/1744666X.2019.1629904 PMC7936577

[B110] Krämer B, Knoll R, Bonaguro L, ToVinh M, Raabe J, Astaburuaga-García R, Schulte-Schrepping J, Kaiser KM, Rieke GJ, Bischoff J, Monin MB, Hoffmeister C, Schlabe S, De Domenico E, Reusch N, Händler K, Reynolds G, Blüthgen N, Hack G, Finnemann C, Nischalke HD, Strassburg CP, Stephenson E, Su Y, Gardner L, Yuan D, Chen D, Goldman J, Rosenstiel P, Schmidt SV, Latz E, Hrusovsky K, Ball AJ, Johnson JM, Koenig PA, Schmidt FI, Haniffa M, Heath JR, Kümmerer BM, Keitel V, Jensen B, Stubbemann P, Kurth F, Sander LE, Sawitzki B, Aschenbrenner AC, Schultze JL, Nattermann J. 2021. Early IFN-α signatures and persistent dysfunction are distinguishing features of NK cells in severe COVID-19. Immunity 54(11):2650–2669.e14. 34592166 10.1016/j.immuni.2021.09.002 PMC8416549

[B111] Krammer F, Smith GJD, Fouchier RAM, Peiris M, Kedzierska K, Doherty PC, Palese P, Shaw ML, Treanor J, Webster RG, García-Sastre A. 2018. Influenza. Nat Rev Dis Primers 4(1):3. 29955068 10.1038/s41572-018-0002-y PMC7097467

[B112] Kumar V. 2020. Toll-like receptors in sepsis-associated cytokine storm and their endogenous negative regulators as future immunomodulatory targets. Int Immunopharmacol 89(Pt B):107087. 10.1016/j.intimp.2020.107087 PMC7550173 33075714

[B113] Lam ET, Eckhardt SG, Messersmith W, Jimeno A, O'Bryant CL, Ramanathan RK, Weiss GJ, Chadha M, Oey A, Ding HT, Culp PA, Keller SF, Zhao VY, Tsao LC, Singhal A, Holen KD, Von Hoff D. 2018. Phase I Study of Enavatuzumab, a first-in-class humanized monoclonal antibody targeting the TWEAK receptor, in patients with advanced solid tumors. Mol Cancer Ther 17(1):215–221. 29054986 10.1158/1535-7163.MCT-17-0330 PMC5752572

[B114] Lanz C, Yángüez E, Andenmatten D, Stertz S. 2015. Swine interferon-inducible transmembrane proteins potently inhibit influenza A virus replication. J Virol 89(1):863–869. 25320322 10.1128/JVI.02516-14 PMC4301123

[B115] Latino I, Gonzalez SF. 2021. Spatio-temporal profile of innate inflammatory cells and mediators during influenza virus infection. Curr Opin Physiol 19:175–186.

[B116] Lauro S, Onesti CE, Righini R, Marchetti P. 2014. The use of bevacizumab in non-small cell lung cancer: an update. Anticancer Res 34(4):1537–1545. 24692680

[B117] Le Goffic R, Pothlichet J, Vitour D, Fujita T, Meurs E, Chignard M, Si-Tahar M. 2007. Cutting Edge: Influenza A virus activates TLR3-dependent inflammatory and RIG-I-dependent antiviral responses in human lung epithelial cells. J Immunol 178(6):3368–3372. 17339430 10.4049/jimmunol.178.6.3368

[B118] Leavis HL, van de Veerdonk FL, Murthy S. 2022. Stimulating severe COVID-19: the potential role of GM-CSF antagonism. Lancet Respir Med 10(3):223–224. 34863335 10.1016/S2213-2600(21)00539-7 PMC8635456

[B119] Lee JS, Park S, Jeong HW, Ahn JY, Choi SJ, Lee H, Choi B, Nam SK, Sa M, Kwon JS, Jeong SJ, Lee HK, Park SH, Park SH, Choi JY, Kim SH, Jung I, Shin EC. 2020. Immunophenotyping of COVID-19 and influenza highlights the role of type I interferons in development of severe COVID-19. Sci Immunol 5(49):eabd1554. 32651212 10.1126/sciimmunol.abd1554 PMC7402635

[B120] Lee N, Wong CK, Chan PK, Chan MC, Wong RY, Lun SW, Ngai KL, Lui GC, Wong BC, Lee SK, Choi KW, Hui DS. 2011. Cytokine response patterns in severe pandemic 2009 H1N1 and seasonal influenza among hospitalized adults. PLoS One 6(10):e26050. 22022504 10.1371/journal.pone.0026050 PMC3192778

[B121] Levin EG, Lustig Y, Cohen C, Fluss R, Indenbaum V, Amit S, Doolman R, Asraf K, Mendelson E, Ziv A, Rubin C, Freedman L, Kreiss Y, Regev-Yochay G. 2021. Waning immune humoral response to BNT162b2 Covid-19 vaccine over 6 months. N Engl J Med 385(24):e84. 34614326 10.1056/NEJMoa2114583 PMC8522797

[B122] Li F. 2016. Structure, function, and evolution of coronavirus spike proteins. Annu Rev Virol 3(1):237–261. 27578435 10.1146/annurev-virology-110615-042301 PMC5457962

[B123] Liao M, Liu Y, Yuan J, Wen Y, Xu G, Zhao J, Cheng L, Li J, Wang X, Wang F, Liu L, Amit I, Zhang S, Zhang Z. 2020. Single-cell landscape of bronchoalveolar immune cells in patients with COVID-19. Nat Med 26(6):842–844. 32398875 10.1038/s41591-020-0901-9

[B124] Lim YX, Ng YL, Tam JP, Liu DX. 2016. Human coronaviruses: a review of virus-host interactions. Diseases 4(3):26. 10.3390/diseases4030026 PMC5456285 28933406

[B125] Limaye AP, Kirby KA, Rubenfeld GD, Leisenring WM, Bulger EM, Neff MJ, Gibran NS, Huang ML, Santo Hayes TK, Corey L, Boeckh M. 2008. Cytomegalovirus reactivation in critically ill immunocompetent patients. JAMA 300(4):413–422. 18647984 10.1001/jama.300.4.413 PMC2774501

[B126] Liu B, Bao L, Wang L, Li F, Wen M, Li H, Deng W, Zhang X, Cao B. 2021. Anti-IFN-γ therapy alleviates acute lung injury induced by severe influenza A (H1N1) pdm09 infection in mice. J Microbiol Immunol Infect 54(3):396–403. 31780358 10.1016/j.jmii.2019.07.009

[B127] Liu Q, Zhou YH, Yang ZQ. 2016. The cytokine storm of severe influenza and development of immunomodulatory therapy. Cell Mol Immunol 13(1):3–10. 26189369 10.1038/cmi.2015.74 PMC4711683

[B128] Liu S, Yan R, Chen B, Pan Q, Chen Y, Hong J, Zhang L, Liu W, Wang S, Chen JL. 2019. Influenza virus-induced robust expression of SOCS3 contributes to excessive production of IL-6. Front Immunol 10:1843. 31474976 10.3389/fimmu.2019.01843 PMC6706793

[B129] Locatelli F, Jordan MB, Allen C, Cesaro S, Rizzari C, Rao A, Degar B, Garrington TP, Sevilla J, Putti M-C, Fagioli F, Ahlmann M, Dapena Diaz J-L, Henry M, De Benedetti F, Grom A, Lapeyre G, Jacqmin P, Ballabio M, de Min C. 2020. Emapalumab in children with primary hemophagocytic lymphohistiocytosis. N Engl J Med 382(19):1811–1822. 32374962 10.1056/NEJMoa1911326

[B130] Lu W, Liu X, Wang T, Liu F, Zhu A, Lin Y, Luo J, Ye F, He J, Zhao J, Li Y, Zhong N. 2021. Elevated MUC1 and MUC5AC mucin protein levels in airway mucus of critical ill COVID-19 patients. J Med Virol 93(2):582–584. 32776556 10.1002/jmv.26406 PMC7436726

[B131] Lucas C, Wong P, Klein J, Castro TBR, Silva J, Sundaram M, Ellingson MK, Mao T, Oh JE, Israelow B, Takahashi T, Tokuyama M, Lu P, Venkataraman A, Park A, Mohanty S, Wang H, Wyllie AL, Vogels CBF, Earnest R, Lapidus S, Ott IM, Moore AJ, Muenker MC, Fournier JB, Campbell M, Odio CD, Casanovas-Massana A, Herbst R, Shaw AC, Medzhitov R, Schulz WL, Grubaugh ND, Dela Cruz C, Farhadian S, Ko AI, Omer SB, Iwasaki A. 2020. Longitudinal analyses reveal immunological misfiring in severe COVID-19. Nature 584(7821):463–469. 32717743 10.1038/s41586-020-2588-y PMC7477538

[B132] Luheshi N, Davies G, Poon E, Wiggins K, McCourt M, Legg J. 2014. Th1 cytokines are more effective than Th2 cytokines at licensing anti-tumour functions in CD40-activated human macrophages in vitro. Eur J Immunol 44(1):162–172. 24114634 10.1002/eji.201343351

[B133] Lukan N. 2020. “Cytokine storm”, not only in COVID-19 patients. Mini-review. Immunol Lett 228:38–44. 33007369 10.1016/j.imlet.2020.09.007 PMC7524442

[B134] Lund JM, Alexopoulou L, Sato A, Karow M, Adams NC, Gale NW, Iwasaki A, Flavell RA. 2004. Recognition of single-stranded RNA viruses by Toll-like receptor 7. Proc Natl Acad Sci U S A 101(15):5598–5603. 15034168 10.1073/pnas.0400937101 PMC397437

[B135] Lustig Y, Sapir E, Regev-Yochay G, Cohen C, Fluss R, Olmer L, Indenbaum V, Mandelboim M, Doolman R, Amit S, Mendelson E, Ziv A, Huppert A, Rubin C, Freedman L, Kreiss Y. 2021. BNT162b2 COVID-19 vaccine and correlates of humoral immune responses and dynamics: a prospective, single-centre, longitudinal cohort study in health-care workers. Lancet Respir Med 9(9):999–1009. 34224675 10.1016/S2213-2600(21)00220-4 PMC8253545

[B136] Lv J, Wang Z, Qu Y, Zhu H, Zhu Q, Tong W, Bao L, Lv Q, Cong J, Li D, Deng W, Yu P, Song J, Tong W-M, Liu J, Liu Y, Qin C, Huang B. 2021. Distinct uptake, amplification, and release of SARS-CoV-2 by M1 and M2 alveolar macrophages. Cell Discov 7(1):24. 33850112 10.1038/s41421-021-00258-1 PMC8043100

[B137] Maniatis NA, Orfanos SE. 2008. The endothelium in acute lung injury/acute respiratory distress syndrome. Curr Opin Crit Care 14(1):22–30. 18195622 10.1097/MCC.0b013e3282f269b9

[B138] Mantlo E, Bukreyeva N, Maruyama J, Paessler S, Huang C. 2020. Antiviral activities of type I interferons to SARS-CoV-2 infection. Antiviral Res 179:104811. 32360182 10.1016/j.antiviral.2020.104811 PMC7188648

[B139] Mantovani A, Locati M, Vecchi A, Sozzani S, Allavena P. 2001. Decoy receptors: a strategy to regulate inflammatory cytokines and chemokines. Trends Immunol 22(6):328–336. 11377293 10.1016/s1471-4906(01)01941-x

[B140] Martin TR, Frevert CW. 2005. Innate immunity in the lungs. Proc Am Thorac Soc 2(5):403–411. 16322590 10.1513/pats.200508-090JS PMC2713330

[B141] Martinez-Sanchez ME, Huerta L, Alvarez-Buylla ER, Villarreal Luján C. 2018. Role of cytokine combinations on CD4+ T cell differentiation, partial polarization, and plasticity: continuous network modeling approach. Front Physiol 9:877. 30127748 10.3389/fphys.2018.00877 PMC6089340

[B142] Masood KI, Yameen M, Ashraf J, Shahid S, Mahmood SF, Nasir A, Nasir N, Jamil B, Ghanchi NK, Khanum I, Razzak SA, Kanji A, Hussain R, M ER, Hasan Z. 2021. Upregulated type I interferon responses in asymptomatic COVID-19 infection are associated with improved clinical outcome. Sci Rep 11(1):22958. 34824360 10.1038/s41598-021-02489-4 PMC8617268

[B143] Matsuyama S, Nao N, Shirato K, Kawase M, Saito S, Takayama I, Nagata N, Sekizuka T, Katoh H, Kato F, Sakata M, Tahara M, Kutsuna S, Ohmagari N, Kuroda M, Suzuki T, Kageyama T, Takeda M. 2020. Enhanced isolation of SARS-CoV-2 by TMPRSS2-expressing cells. Proc Natl Acad Sci U S A 117(13):7001–7003. 32165541 10.1073/pnas.2002589117 PMC7132130

[B144] Matzinger P, Kamala T. 2011. Tissue-based class control: the other side of tolerance. Nat Rev Immunol 11(3):221–230. 21350581 10.1038/nri2940

[B145] Maucourant C, Filipovic I, Ponzetta A, Aleman S, Cornillet M, Hertwig L, Strunz B, Lentini A, Reinius B, Brownlie D, Cuapio A, Ask EH, Hull RM, Haroun-Izquierdo A, Schaffer M, Klingström J, Folkesson E, Buggert M, Sandberg JK, Eriksson LI, Rooyackers O, Ljunggren H-G, Malmberg K-J, Michaëlsson J, Marquardt N, Hammer Q, Strålin K, Björkström NK. 2020. Natural killer cell immunotypes related to COVID-19 disease severity. Sci Immunol 5(50):eabd6832. 32826343 10.1126/sciimmunol.abd6832 PMC7665314

[B146] McAuley JL, Corcilius L, Tan HX, Payne RJ, McGuckin MA, Brown LE. 2017. The cell surface mucin MUC1 limits the severity of influenza A virus infection. Mucosal Immunol 10(6):1581–1593. 28327617 10.1038/mi.2017.16

[B147] Meduri GU, Kohler G, Headley S, Tolley E, Stentz F, Postlethwaite A. 1995. Inflammatory cytokines in the BAL of patients with ARDS. Persistent elevation over time predicts poor outcome. Chest 108(5):1303–1314. 7587434 10.1378/chest.108.5.1303

[B148] Mehta P, McAuley DF, Brown M, Sanchez E, Tattersall RS, Manson JJ, Hlh Across Speciality Collaboration UK. 2020. COVID-19: consider cytokine storm syndromes and immunosuppression. Lancet 395(10229):1033–1034. 32192578 10.1016/S0140-6736(20)30628-0 PMC7270045

[B149] Meischel T, Fritzlar S, Villalon-Letelier F, Tessema MB, Brooks AG, Reading PC, Londrigan SL. 2021. IFITM proteins that restrict the early stages of respiratory virus infection do not influence late-stage replication. J Virol 95(20):e0083721. 34319159 10.1128/JVI.00837-21 PMC8475524

[B150] Menten P, Wuyts A, Van Damme J. 2002. Macrophage inflammatory protein-1. Cytokine Growth Factor Rev 13(6):455–481. 12401480 10.1016/s1359-6101(02)00045-x

[B151] Menzies-Gow A, Corren J, Bourdin A, Chupp G, Israel E, Wechsler ME, Brightling CE, Griffiths JM, Hellqvist Å, Bowen K, Kaur P, Almqvist G, Ponnarambil S, Colice G. 2021. Tezepelumab in adults and adolescents with severe, uncontrolled asthma. N Engl J Med 384(19):1800–1809. 33979488 10.1056/NEJMoa2034975

[B152] Mera S, Tatulescu D, Cismaru C, Bondor C, Slavcovici A, Zanc V, Carstina D, Oltean M. 2011. Multiplex cytokine profiling in patients with sepsis. APMIS 119(2):155–163. 21208283 10.1111/j.1600-0463.2010.02705.x

[B153] Merad M MJ. 2020. Pathological inflammation in patients with COVID-19: a key role for monocytes and macrophages. Nat Rev Immunol 20(6):355–362. 32376901 10.1038/s41577-020-0331-4 PMC7201395

[B154] Mo X, Jian W, Su Z, Chen M, Peng H, Peng P, Lei C, Chen R, Zhong N, Li S. 2020. Abnormal pulmonary function in COVID-19 patients at time of hospital discharge. Eur Respir J 55(6):2001217. 32381497 10.1183/13993003.01217-2020 PMC7236826

[B155] Moran TM, Park H, Fernandez-Sesma A, Schulman JL. 1999. Th2 responses to inactivated influenza virus can be converted to Th1 responses and facilitate recovery from heterosubtypic virus infection. J Infect Dis 180(3):579–585. 10438342 10.1086/314952

[B156] Mori S, Ueki Y, Hirakata N, Oribe M, Hidaka T, Oishi K. 2012. Impact of tocilizumab therapy on antibody response to influenza vaccine in patients with rheumatoid arthritis. Ann Rheum Dis 71(12):2006–2010. 22887851 10.1136/annrheumdis-2012-201950 PMC3595981

[B157] Mudd PA, Crawford JC, Turner JS, Souquette A, Reynolds D, Bender D, Bosanquet JP, Anand NJ, Striker DA, Martin RS, Boon ACM, House SL, Remy KE, Hotchkiss RS, Presti RM, O'Halloran JA, Powderly WG, Thomas PG, Ellebedy AH. 2020. Distinct inflammatory profiles distinguish COVID-19 from influenza with limited contributions from cytokine storm. Sci Adv 6(50):eabe3024. 33187979 10.1126/sciadv.abe3024 PMC7725462

[B158] Nakae H, Endo S, Inada K, Takakuwa T, Kasai T. 1996. Changes in adhesion molecule levels in sepsis. Res Commun Mol Pathol Pharmacol 91(3):329–338. 8829772

[B159] Nakanishi K. 2018. Unique action of interleukin-18 on T cells and other immune cells. Front Immunol 9:763. 29731751 10.3389/fimmu.2018.00763 PMC5920033

[B160] Nakanishi K, Yoshimoto T, Tsutsui H, Okamura H. 2001. Interleukin-18 regulates both Th1 and Th2 responses. Annu Rev Immunol 19:423–474. 11244043 10.1146/annurev.immunol.19.1.423

[B161] Naranbhai V, Nathan A, Kaseke C, Berrios C, Khatri A, Choi S, Getz MA, Tano-Menka R, Ofoman O, Gayton A, Senjobe F, Denis KJS, Lam EC, Garcia-Beltran WF, Balazs AB, Walker BD, Iafrate AJ, Gaiha GD. 2022. T cell reactivity to the SARS-CoV-2 Omicron variant is preserved in most but not all prior infected and vaccinated individuals. medRxiv [Epub ahead of print]; DOI: 10.1101/2022.01.04.21268586.

[B162] Novel Swine-Origin Influenza AVIT, Dawood FS, Jain S, Finelli L, Shaw MW, Lindstrom S, Garten RJ, Gubareva LV, Xu X, Bridges CB, Uyeki TM. 2009. Emergence of a novel swine-origin influenza A (H1N1) virus in humans. N Engl J Med 360(25):2605–2615. 19423869 10.1056/NEJMoa0903810

[B163] Okada M, Kitahara M, Kishimoto S, Matsuda T, Hirano T, Kishimoto T. 1988. IL-6/BSF-2 functions as a killer helper factor in the in vitro induction of cytotoxic T cells. J Immunol 141(5):1543–1549. 3261754

[B164] Okeke EB, Uzonna JE. 2019. The pivotal role of regulatory T cells in the regulation of innate immune cells. Front Immunol 10:680. 31024539 10.3389/fimmu.2019.00680 PMC6465517

[B165] Olbei M, Hautefort I, Modos D, Treveil A, Poletti M, Gul L, Shannon-Lowe CD, Korcsmaros T. 2021. SARS-CoV-2 causes a different cytokine response compared to other cytokine storm-causing respiratory viruses in severely Ill patients. Front Immunol 12:629193. 33732251 10.3389/fimmu.2021.629193 PMC7956943

[B166] Opal SM. 2011. The evolution of the understanding of sepsis, infection, and the host response: a brief history. Crit Care Nurs Clin North Am 23(1):1–27. 21316565 10.1016/j.ccell.2010.12.001

[B167] Ou X, Liu Y, Lei X, Li P, Mi D, Ren L, Guo L, Guo R, Chen T, Hu J, Xiang Z, Mu Z, Chen X, Chen J, Hu K, Jin Q, Wang J, Qian Z. 2020. Characterization of spike glycoprotein of SARS-CoV-2 on virus entry and its immune cross-reactivity with SARS-CoV. Nat Commun 11(1):1620. 32221306 10.1038/s41467-020-15562-9 PMC7100515

[B168] Pan P, Shen M, Yu Z, Ge W, Chen K, Tian M, Xiao F, Wang Z, Wang J, Jia Y, Wang W, Wan P, Zhang J, Chen W, Lei Z, Chen X, Luo Z, Zhang Q, Xu M, Li G, Li Y, Wu J. 2021. SARS-CoV-2 N protein promotes NLRP3 inflammasome activation to induce hyperinflammation. Nat Commun 12(1):4664. 34341353 10.1038/s41467-021-25015-6 PMC8329225

[B169] Pang J, Xu F, Aondio G, Li Y, Fumagalli A, Lu M, Valmadre G, Wei J, Bian Y, Canesi M, Damiani G, Zhang Y, Yu D, Chen J, Ji X, Sui W, Wang B, Wu S, Kovacs A, Revera M, Wang H, Jing X, Zhang Y, Chen Y, Cao Y. 2021. Efficacy and tolerability of bevacizumab in patients with severe Covid-19. Nat Commun 12(1):814. 33547300 10.1038/s41467-021-21085-8 PMC7864918

[B170] Paquette SG, Banner D, Zhao Z, Fang Y, Huang SS, León AJ, Ng DC, Almansa R, Martin-Loeches I, Ramirez P, Socias L, Loza A, Blanco J, Sansonetti P, Rello J, Andaluz D, Shum B, Rubino S, de Lejarazu RO, Tran D, Delogu G, Fadda G, Krajden S, Rubin BB, Bermejo-Martin JF, Kelvin AA, Kelvin DJ. 2012. Interleukin-6 is a potential biomarker for severe pandemic H1N1 influenza A infection. PLoS One 7(6):e38214. 22679491 10.1371/journal.pone.0038214 PMC3367995

[B171] Patterson BK, Seethamraju H, Dhody K, Corley MJ, Kazempour K, Lalezari JP, Pang AP, Sugai C, Francisco EB, Pise A, Rodrigues H, Ryou M, Wu HL, Webb GM, Park BS, Kelly S, Pourhassan N, Lelic A, Kdouh L, Herrera M, Hall E, Aklin E, Ndhlovu L, Sacha JB. 2020. Disruption of the CCL5/RANTES-CCR5 pathway restores immune homeostasis and reduces plasma viral load in critical COVID-19. medRxiv [Epub ahead of print]; DOI: 10.1101/2020.05.02.20084673.

[B172] Peckham H, de Gruijter NM, Raine C, Radziszewska A, Ciurtin C, Wedderburn LR, Rosser EC, Webb K, Deakin CT. 2020. Male sex identified by global COVID-19 meta-analysis as a risk factor for death and ITU admission. Nat Commun 11(1):6317. 33298944 10.1038/s41467-020-19741-6 PMC7726563

[B173] Pennica D, Lam VT, Weber RF, Kohr WJ, Basa LJ, Spellman MW, Ashkenazi A, Shire SJ, Goeddel DV. 1993. Biochemical characterization of the extracellular domain of the 75-kilodalton tumor necrosis factor receptor. Biochemistry 32(12):3131–3138. 8384489 10.1021/bi00063a027

[B174] Perez-Padilla R, de la Rosa-Zamboni D, Ponce de Leon S, Hernandez M, Quiñones-Falconi F, Bautista E, Ramirez-Venegas A, Rojas-Serrano J, Ormsby CE, Corrales A, Higuera A, Mondragon E, Cordova-Villalobos JA. 2009. Pneumonia and respiratory failure from swine-origin influenza A (H1N1) in Mexico. N Engl J Med 361(7):680–689. 19564631 10.1056/NEJMoa0904252

[B175] Pinto RA, Arredondo SM, Bono MR, Gaggero AA, Díaz PV. 2006. T helper 1/T helper 2 cytokine imbalance in respiratory syncytial virus infection is associated with increased endogenous plasma cortisol. Pediatrics 117(5):e878–e886. 16618789 10.1542/peds.2005-2119

[B176] Plante JA, Plante KS, Gralinski LE, Beall A, Ferris MT, Bottomly D, Green R, McWeeney SK, Heise MT, Baric RS, Menachery VD. 2020. Mucin 4 protects female mice from coronavirus pathogenesis. bioRxiv:2020.02.19.957118.

[B177] Porter DL, Hwang WT, Frey NV, Lacey SF, Shaw PA, Loren AW, Bagg A, Marcucci KT, Shen A, Gonzalez V, Ambrose D, Grupp SA, Chew A, Zheng Z, Milone MC, Levine BL, Melenhorst JJ, June CH. 2015. Chimeric antigen receptor T cells persist and induce sustained remissions in relapsed refractory chronic lymphocytic leukemia. Sci Transl Med 7(303):303ra139. 10.1126/scitranslmed.aac5415 PMC5909068 26333935

[B178] Poyiadji N, Shahin G, Noujaim D, Stone M, Patel S, Griffith B. 2020. COVID-19-associated Acute Hemorrhagic Necrotizing Encephalopathy: Imaging Features. Radiology 296(2):E119–E120. 32228363 10.1148/radiol.2020201187 PMC7233386

[B179] Price CC, Altice FL, Shyr Y, Koff A, Pischel L, Goshua G, Azar MM, McManus D, Chen SC, Gleeson SE, Britto CJ, Azmy V, Kaman K, Gaston DC, Davis M, Burrello T, Harris Z, Villanueva MS, Aoun-Barakat L, Kang I, Seropian S, Chupp G, Bucala R, Kaminski N, Lee AI, LoRusso PM, Topal JE, Dela Cruz C, Malinis M. 2020. Tocilizumab treatment for cytokine release syndrome in hospitalized patients with coronavirus disease 2019: survival and clinical outcomes. Chest 158(4):1397–1408. 32553536 10.1016/j.chest.2020.06.006 PMC7831876

[B180] Pum A, Ennemoser M, Adage T, Kungl AJ. 2021. Cytokines and chemokines in SARS-CoV-2 infections-therapeutic strategies targeting cytokine storm. Biomolecules 11(1):91. 33445810 10.3390/biom11010091 PMC7828218

[B181] Remy KE, Mazer M, Striker DA, Ellebedy AH, Walton AH, Unsinger J, Blood TM, Mudd PA, Yi DJ, Mannion DA, Osborne DF, Martin RS, Anand NJ, Bosanquet JP, Blood J, Drewry AM, Caldwell CC, Turnbull IR, Brakenridge SC, Moldwawer LL, Hotchkiss RS. 2020. Severe immunosuppression and not a cytokine storm characterizes COVID-19 infections. JCI Insight 5(17):e140329. 10.1172/jci.insight.140329 PMC7526441 32687484

[B182] Ren R, Wu S, Cai J, Yang Y, Ren X, Feng Y, Chen L, Qin B, Xu C, Yang H, Song Z, Tian D, Hu Y, Zhou X, Meng G. 2017. The H7N9 influenza A virus infection results in lethal inflammation in the mammalian host via the NLRP3-caspase-1 inflammasome. Sci Rep 7(1):7625. 28790324 10.1038/s41598-017-07384-5 PMC5548739

[B183] Rendón-Ramirez EJ, Ortiz-Stern A, Martinez-Mejia C, Salinas-Carmona MC, Rendon A, Mata-Tijerina VL, Rosas-Taraco AG. 2015. TGF-β blood levels distinguish between influenza A (H1N1)pdm09 virus sepsis and sepsis due to other forms of community-acquired pneumonia. Viral Immunol 28(5):248–254. 25923384 10.1089/vim.2014.0123 PMC4486447

[B184] Reusch N, De Domenico E, Bonaguro L, Schulte-Schrepping J, Baßler K, Schultze JL, Aschenbrenner AC. 2021. Neutrophils in COVID-19. Front Immunol 12:652470. 33841435 10.3389/fimmu.2021.652470 PMC8027077

[B185] Reynolds D, Vazquez Guillamet C, Day A, Borcherding N, Vazquez Guillamet R, Choreño-Parra JA, House SL, O'Halloran JA, Zúñiga J, Ellebedy AH, Byers DE, Mudd PA. 2021. Comprehensive immunologic evaluation of bronchoalveolar lavage samples from human patients with moderate and severe seasonal influenza and severe COVID-19. J Immunol 207(5):1229–1238. 34348975 10.4049/jimmunol.2100294 PMC8387368

[B186] Roblek M, Strutzmann E, Zankl C, Adage T, Heikenwalder M, Atlic A, Weis R, Kungl A, Borsig L. 2016. Targeting of CCL2-CCR2-glycosaminoglycan axis using a CCL2 decoy protein attenuates metastasis through inhibition of tumor cell seeding. Neoplasia 18(1):49–59. 26806351 10.1016/j.neo.2015.11.013 PMC4735630

[B187] Rodrigues TS, de Sá KSG, Ishimoto AY, Becerra A, Oliveira S, Almeida L, Gonçalves AV, Perucello DB, Andrade WA, Castro R, Veras FP, Toller-Kawahisa JE, Nascimento DC, de Lima MHF, Silva CMS, Caetite DB, Martins RB, Castro IA, Pontelli MC, de Barros FC, do Amaral NB, Giannini MC, Bonjorno LP, Lopes MIF, Santana RC, Vilar FC, Auxiliadora-Martins M, Luppino-Assad R, de Almeida SCL, de Oliveira FR, Batah SS, Siyuan L, Benatti MN, Cunha TM, Alves-Filho JC, Cunha FQ, Cunha LD, Frantz FG, Kohlsdorf T, Fabro AT, Arruda E, de Oliveira RDR, Louzada-Junior P, Zamboni DS. 2021. Inflammasomes are activated in response to SARS-CoV-2 infection and are associated with COVID-19 severity in patients. J Exp Med 218(3):e20201707. 33231615 10.1084/jem.20201707 PMC7684031

[B188] Rohaim MA, Al-Natour MQ, Abdelsabour MA, El Naggar RF, Madbouly YM, Ahmed KA, Munir M. 2021. Transgenic chicks expressing interferon-inducible transmembrane protein 1 (IFITM1) restrict highly pathogenic H5N1 influenza viruses. Int J Mol Sci 22(16):8456. 34445163 10.3390/ijms22168456 PMC8395118

[B189] Rosa BA, Ahmed M, Singh DK, Choreño-Parra JA, Cole J, Jiménez-Álvarez LA, Rodríguez-Reyna TS, Singh B, Gonzalez O, Carrion R, Schlesinger LS, Martin J, Zúñiga J, Mitreva M, Kaushal D, Khader SA. 2021. IFN signaling and neutrophil degranulation transcriptional signatures are induced during SARS-CoV-2 infection. Commun Biol 4(1):290. 33674719 10.1038/s42003-021-01829-4 PMC7935909

[B190] Rosas-Ballina M, Olofsson PS, Ochani M, Valdés-Ferrer SI, Levine YA, Reardon C, Tusche MW, Pavlov VA, Andersson U, Chavan S, Mak TW, Tracey KJ. 2011. Acetylcholine-synthesizing T cells relay neural signals in a vagus nerve circuit. Science 334(6052):98–101. 21921156 10.1126/science.1209985 PMC4548937

[B191] Roy MG, Livraghi-Butrico A, Fletcher AA, McElwee MM, Evans SE, Boerner RM, Alexander SN, Bellinghausen LK, Song AS, Petrova YM, Tuvim MJ, Adachi R, Romo I, Bordt AS, Bowden MG, Sisson JH, Woodruff PG, Thornton DJ, Rousseau K, De la Garza MM, Moghaddam SJ, Karmouty-Quintana H, Blackburn MR, Drouin SM, Davis CW, Terrell KA, Grubb BR, O'Neal WK, Flores SC, Cota-Gomez A, Lozupone CA, Donnelly JM, Watson AM, Hennessy CE, Keith RC, Yang IV, Barthel L, Henson PM, Janssen WJ, Schwartz DA, Boucher RC, Dickey BF, Evans CM. 2014. Muc5b is required for airway defence. Nature 505(7483):412–416. 24317696 10.1038/nature12807 PMC4001806

[B192] Saas P, Boucraut J, Walker PR, Quiquerez AL, Billot M, Desplat-Jego S, Chicheportiche Y, Dietrich PY. 2000. TWEAK stimulation of astrocytes and the proinflammatory consequences. Glia 32(1):102–107. 10975915

[B193] Saito S, Uchino S, Hayakawa M, Yamakawa K, Kudo D, Iizuka Y, Sanui M, Takimoto K, Mayumi T, Sasabuchi Y. 2019. Epidemiology of disseminated intravascular coagulation in sepsis and validation of scoring systems. J Crit Care 50:23–30. 30471557 10.1016/j.jcrc.2018.11.009

[B194] Sarkar M, Saha S. 2020. Structural insight into the role of novel SARS-CoV-2 E protein: a potential target for vaccine development and other therapeutic strategies. PLoS One 15(8):e0237300. 32785274 10.1371/journal.pone.0237300 PMC7423102

[B195] Schoeman D, Fielding BC. 2019. Coronavirus envelope protein: current knowledge. Virol J 16(1):69. 31133031 10.1186/s12985-019-1182-0 PMC6537279

[B196] Schönfelder K, Breuckmann K, Elsner C, Dittmer U, Fistera D, Herbstreit F, Risse J, Schmidt K, Sutharsan S, Taube C, Jöckel KH, Siffert W, Kribben A, Möhlendick B. 2021. The influence of IFITM3 polymorphisms on susceptibility to SARS-CoV-2 infection and severity of COVID-19. Cytokine 142:155492. 33711707 10.1016/j.cyto.2021.155492 PMC7936555

[B197] Schouten M, Wiersinga WJ, Levi M, van der Poll T. 2008. Inflammation, endothelium, and coagulation in sepsis. J Leukoc Biol 83(3):536–545. 18032692 10.1189/jlb.0607373

[B198] Schulte W, Bernhagen J, Bucala R. 2013. Cytokines in sepsis: potent immunoregulators and potential therapeutic targets—an updated view. Mediat Inflamm 2013:165974. 10.1155/2013/165974 PMC3703895 23853427

[B199] Sereno M, Jimenez-Gordo AM, Baena-Espinar J, Aguado C, Mielgo X, Pertejo A, Álvarez-Álvarez R, Sánchez A, López JL, Molina R, López-Alfonso A, Hernández B, Chiara LE, Martín AM, López-Martín A, Dorta M, Collazo-Lorduy A, Casado E, de Molina AR, Colmenarejo G. 2021. A Multicenter analysis of the outcome of cancer patients with neutropenia and COVID-19 optionally treated with granulocyte-colony stimulating factor (G-CSF): a comparative analysis. Cancers (Basel) 13(16):4205. 34439359 10.3390/cancers13164205 PMC8391975

[B200] Shaan Lakshmanappa Y, Elizaldi SR, Roh JW, Schmidt BA, Carroll TD, Weaver KD, Smith JC, Verma A, Deere JD, Dutra J, Stone M, Franz S, Sammak RL, Olstad KJ, Rachel Reader J, Ma Z-M, Nguyen NK, Watanabe J, Usachenko J, Immareddy R, Yee JL, Weiskopf D, Sette A, Hartigan-O'Connor D, McSorley SJ, Morrison JH, Tran NK, Simmons G, Busch MP, Kozlowski PA, Van Rompay KKA, Miller CJ, Iyer SS. 2021. SARS-CoV-2 induces robust germinal center CD4 T follicular helper cell responses in rhesus macaques. Nat Commun 12(1):541. 33483492 10.1038/s41467-020-20642-x PMC7822826

[B201] Sheahan T, Morrison TE, Funkhouser W, Uematsu S, Akira S, Baric RS, Heise MT. 2008. MyD88 is required for protection from lethal infection with a mouse-adapted SARS-CoV. PLoS Pathog 4(12):e1000240. 19079579 10.1371/journal.ppat.1000240 PMC2587915

[B202] Shekhar S, Peng Y, Wang S, Yang X. 2018. CD103+ lung dendritic cells (LDCs) induce stronger Th1/Th17 immunity to a bacterial lung infection than CD11b(hi) LDCs. Cell Mol Immunol 15(4):377–387. 28194020 10.1038/cmi.2016.68 PMC6052831

[B203] Shen XF, Cao K, Jiang JP, Guan WX, Du JF. 2017. Neutrophil dysregulation during sepsis: an overview and update. J Cell Mol Med 21(9):1687–1697. 28244690 10.1111/jcmm.13112 PMC5571534

[B204] Shi CS, Nabar NR, Huang NN, Kehrl JH. 2019. SARS-Coronavirus Open Reading Frame-8b triggers intracellular stress pathways and activates NLRP3 inflammasomes. Cell Death Discov 5:101. 31231549 10.1038/s41420-019-0181-7 PMC6549181

[B205] Shrestha SS, Swerdlow DL, Borse RH, Prabhu VS, Finelli L, Atkins CY, Owusu-Edusei K, Bell B, Mead PS, Biggerstaff M, Brammer L, Davidson H, Jernigan D, Jhung MA, Kamimoto LA, Merlin TL, Nowell M, Redd SC, Reed C, Schuchat A, Meltzer MI. 2011. Estimating the burden of 2009 pandemic influenza A (H1N1) in the United States (April 2009-April 2010). Clin Infect Dis 52 (Suppl 1):S75–S82. 21342903 10.1093/cid/ciq012

[B206] Sims JT, Krishnan V, Chang CY, Engle SM, Casalini G, Rodgers GH, Bivi N, Nickoloff BJ, Konrad RJ, de Bono S, Higgs RE, Benschop RJ, Ottaviani S, Cardoso A, Nirula A, Corbellino M, Stebbing J. 2021. Characterization of the cytokine storm reflects hyperinflammatory endothelial dysfunction in COVID-19. J Allergy Clin Immunol 147(1):107–111. 32920092 10.1016/j.jaci.2020.08.031 PMC7488591

[B207] Singer M, Deutschman CS, Seymour CW, Shankar-Hari M, Annane D, Bauer M, Bellomo R, Bernard GR, Chiche JD, Coopersmith CM, Hotchkiss RS, Levy MM, Marshall JC, Martin GS, Opal SM, Rubenfeld GD, van der Poll T, Vincent JL, Angus DC. 2016. The third international consensus definitions for sepsis and septic shock (Sepsis-3). JAMA 315(8):801–810. 26903338 10.1001/jama.2016.0287 PMC4968574

[B208] Smith SE, Gibson MS, Wash RS, Ferrara F, Wright E, Temperton N, Kellam P, Fife M. 2013. Chicken interferon-inducible transmembrane protein 3 restricts influenza viruses and lyssaviruses in vitro. J Virol 87(23):12957–12966. 24067955 10.1128/JVI.01443-13 PMC3838109

[B209] Stadlbauer D, Zhu X, McMahon M, Turner JS, Wohlbold TJ, Schmitz AJ, Strohmeier S, Yu W, Nachbagauer R, Mudd PA, Wilson IA, Ellebedy AH, Krammer F. 2019. Broadly protective human antibodies that target the active site of influenza virus neuraminidase. Science 366(6464):499–504. 31649200 10.1126/science.aay0678 PMC7105897

[B210] Stepp SE, Dufourcq-Lagelouse R, Le Deist F, Bhawan S, Certain S, Mathew PA, Henter JI, Bennett M, Fischer A, de Saint Basile G, Kumar V. 1999. Perforin gene defects in familial hemophagocytic lymphohistiocytosis. Science 286(5446):1957–1959. 10583959 10.1126/science.286.5446.1957

[B211] Stravalaci M, Pagani I, Paraboschi EM, Pedotti M, Doni A, Scavello F, Mapelli SN, Sironi M, Perucchini C, Varani L, Matkovic M, Cavalli A, Cesana D, Gallina P, Pedemonte N, Capurro V, Clementi N, Mancini N, Invernizzi P, Bayarri-Olmos R, Garred P, Rappuoli R, Duga S, Bottazzi B, Uguccioni M, Asselta R, Vicenzi E, Mantovani A, Garlanda C. 2022. Recognition and inhibition of SARS-CoV-2 by humoral innate immunity pattern recognition molecules. Nat Immunol 23(2):275–286. 35102342 10.1038/s41590-021-01114-w

[B212] Surbatovic M, Popovic N, Vojvodic D, Milosevic I, Acimovic G, Stojicic M, Veljovic M, Jevdjic J, Djordjevic D, Radakovic S. 2015. Cytokine profile in severe gram-positive and gram-negative abdominal sepsis. Sci Rep 5(1):11355. 26079127 10.1038/srep11355 PMC4468818

[B213] Taha M, Sharma A, Soubani A. 2020. Clinical deterioration during neutropenia recovery after G-CSF therapy in patient with COVID-19. Respir Med Case Rep 31:101231. 32999856 10.1016/j.rmcr.2020.101231 PMC7515585

[B214] Tang X, Du R-H, Wang R, Cao T-Z, Guan L-L, Yang C-Q, Zhu Q, Hu M, Li X-Y, Li Y, Liang L-R, Tong Z-H, Sun B, Peng P, Shi H-Z. 2020. Comparison of Hospitalized Patients With ARDS Caused by COVID-19 and H1N1. Chest 158(1):195–205. 32224074 10.1016/j.chest.2020.03.032 PMC7151343

[B215] Temann UA, Ray P, Flavell RA. 2002. Pulmonary overexpression of IL-9 induces Th2 cytokine expression, leading to immune pathology. J Clin Invest 109(1):29–39. 11781348 10.1172/JCI13696 PMC150821

[B216] Theofilopoulos AN, Baccala R, Beutler B, Kono DH. 2005. Type I interferons (alpha/beta) in immunity and autoimmunity. Annu Rev Immunol 23:307–336. 15771573 10.1146/annurev.immunol.23.021704.115843

[B217] Thomas JM, Pos Z, Reinboth J, Wang RY, Wang E, Frank GM, Lusso P, Trinchieri G, Alter HJ, Marincola FM, Thomas E. 2014. Differential responses of plasmacytoid dendritic cells to influenza virus and distinct viral pathogens. J Virol 88(18):10758–10766. 25008918 10.1128/JVI.01501-14 PMC4178854

[B218] Thomas M, Mani RS, Philip M, Adhikary R, Joshi S, Revadi SS, Buggi S, Desai A, Vasanthapuram R. 2017. Proinflammatory chemokines are major mediators of exuberant immune response associated with Influenza A (H1N1) pdm09 virus infection. J Med Virol 89(8):1373–1381. 28198028 10.1002/jmv.24781

[B219] Thomas S. 2020. The Structure of the Membrane Protein of SARS-CoV-2 Resembles the Sugar Transporter SemiSWEET. Pathog Immun 5(1):342–363. 33154981 10.20411/pai.v5i1.377 PMC7608487

[B220] Thompson AJ, Paulson JC. 2021. Adaptation of influenza viruses to human airway receptors. J Biol Chem 296:100017. 33144323 10.1074/jbc.REV120.013309 PMC7948470

[B221] Tjan LH, Furukawa K, Nagano T, Kiriu T, Nishimura M, Arii J, Hino Y, Iwata S, Nishimura Y, Mori Y. 2021. Early differences in cytokine production by severity of coronavirus disease 2019. J Infect Dis 223(7):1145–1149. 33411935 10.1093/infdis/jiab005 PMC7928883

[B222] Tong M, Xiong Y, Zhu C, Xu H, Zheng Q, Jiang Y, Zou L, Xiao X, Chen F, Yan X, Hu C, Zhu Y. 2021. Serum surfactant protein D in COVID-19 is elevated and correlated with disease severity. BMC Infect Dis 21(1):737. 34344306 10.1186/s12879-021-06447-3 PMC8329621

[B223] Torgersen C, Moser P, Luckner G, Mayr V, Jochberger S, Hasibeder WR, Dünser MW. 2009. Macroscopic postmortem findings in 235 surgical intensive care patients with sepsis. Anesth Analg 108(6):1841–1847. 19448210 10.1213/ane.0b013e318195e11d

[B224] Totura AL, Whitmore A, Agnihothram S, Schafer A, Katze MG, Heise MT, Baric RS. 2015. Toll-like receptor 3 signaling via TRIF contributes to a protective innate immune response to severe acute respiratory syndrome coronavirus infection. mBio 6(3):e00638-15. 26015500 10.1128/mBio.00638-15 PMC4447251

[B225] Turner JS, Kim W, Kalaidina E, Goss CW, Rauseo AM, Schmitz AJ, Hansen L, Haile A, Klebert MK, Pusic I, O'Halloran JA, Presti RM, Ellebedy AH. 2021a. SARS-CoV-2 infection induces long-lived bone marrow plasma cells in humans. Nature 595(7867):421–425. 34030176 10.1038/s41586-021-03647-4

[B226] Turner JS, Lei T, Schmitz AJ, Day A, Choreño-Parra JA, Jiménez-Alvarez L, Cruz-Lagunas A, House SL, Zúñiga J, Ellebedy AH, Mudd PA. 2020a. Impaired cellular immune responses during the first week of severe acute influenza infection. J Infect Dis 222(7):1235–1244. 32369589 10.1093/infdis/jiaa226 PMC7768688

[B227] Turner JS, O'Halloran JA, Kalaidina E, Kim W, Schmitz AJ, Zhou JQ, Lei T, Thapa M, Chen RE, Case JB, Amanat F, Rauseo AM, Haile A, Xie X, Klebert MK, Suessen T, Middleton WD, Shi P-Y, Krammer F, Teefey SA, Diamond MS, Presti RM, Ellebedy AH. 2021b. SARS-CoV-2 mRNA vaccines induce persistent human germinal centre responses. Nature 596(7870):109–113. 34182569 10.1038/s41586-021-03738-2 PMC8935394

[B228] Turner JS, Zhou JQ, Han J, Schmitz AJ, Rizk AA, Alsoussi WB, Lei T, Amor M, McIntire KM, Meade P, Strohmeier S, Brent RI, Richey ST, Haile A, Yang YR, Klebert MK, Suessen T, Teefey S, Presti RM, Krammer F, Kleinstein SH, Ward AB, Ellebedy AH. 2020b. Human germinal centres engage memory and naive B cells after influenza vaccination. Nature 586(7827):127–132. 32866963 10.1038/s41586-020-2711-0 PMC7566073

[B229] Ulloa L, Doody J, Massagué J. 1999. Inhibition of transforming growth factor-beta/SMAD signalling by the interferon-gamma/STAT pathway. Nature 397(6721):710–713. 10067896 10.1038/17826

[B230] Ungar B, Glickman JW, Golant AK, Dubin C, Marushchak O, Gontzes A, Mikhaylov D, Singer GK, Baum D, Wei N, Sanin A, Gruenstein D, Lebwohl MG, Pavel AB, Guttman-Yassky E. 2022. COVID-19 symptoms are attenuated in moderate-to-severe atopic dermatitis patients treated with dupilumab. J Allergy Clin Immunol 10(1):134–142. 10.1016/j.jaip.2021.10.050 PMC8558098 34737108

[B231] van der Poll T, van Deventer SJ. 1999. Cytokines and anticytokines in the pathogenesis of sepsis. Infect Dis Clin North Am 13(2):413–426, ix. 10340175 10.1016/s0891-5520(05)70083-0

[B232] Vanderbeke L, Van Mol P, Van Herck Y, De Smet F, Humblet-Baron S, Martinod K, Antoranz A, Arijs I, Boeckx B, Bosisio FM, Casaer M, Dauwe D, De Wever W, Dooms C, Dreesen E, Emmaneel A, Filtjens J, Gouwy M, Gunst J, Hermans G, Jansen S, Lagrou K, Liston A, Lorent N, Meersseman P, Mercier T, Neyts J, Odent J, Panovska D, Penttila PA, Pollet E, Proost P, Qian J, Quintelier K, Raes J, Rex S, Saeys Y, Sprooten J, Tejpar S, Testelmans D, Thevissen K, Van Buyten T, Vandenhaute J, Van Gassen S, Velásquez Pereira LC, Vos R, Weynand B, Wilmer A, Yserbyt J, Garg AD, Matthys P, Wouters C, Lambrechts D, Wauters E, Wauters J. 2021. Monocyte-driven atypical cytokine storm and aberrant neutrophil activation as key mediators of COVID-19 disease severity. Nat Commun 12(1):4117. 34226537 10.1038/s41467-021-24360-w PMC8257697

[B233] Vaz de Paula CB, de Azevedo MLV, Nagashima S, Martins APC, Malaquias MAS, Miggiolaro AFRdS, da Silva Motta Júnior J, Avelino G, do Carmo LAP, Carstens LB, de Noronha L. 2020. IL-4/IL-13 remodeling pathway of COVID-19 lung injury. Sci Rep 10(1):18689. 33122784 10.1038/s41598-020-75659-5 PMC7596721

[B234] Velavan TP, Pallerla SR, Rüter J, Augustin Y, Kremsner PG, Krishna S, Meyer CG. 2021. Host genetic factors determining COVID-19 susceptibility and severity. EBioMedicine 72:103629. 34655949 10.1016/j.ebiom.2021.103629 PMC8512556

[B235] Vietzen H, Zoufaly A, Traugott M, Aberle J, Aberle SW, Puchhammer-Stöckl E. 2021. Deletion of the NKG2C receptor encoding KLRC2 gene and HLA-E variants are risk factors for severe COVID-19. Genet Med 23(5):963–967. 33500568 10.1038/s41436-020-01077-7 PMC7835668

[B236] Villar J, Ferrando C, Martinez D, Ambros A, Munoz T, Soler JA, Aguilar G, Alba F, Gonzalez-Higueras E, Conesa LA, Martin-Rodriguez C, Diaz-Dominguez FJ, Serna-Grande P, Rivas R, Ferreres J, Belda J, Capilla L, Tallet A, Anon JM, Fernandez RL, Gonzalez-Martin JM, dexamethasone in An. 2020. Dexamethasone treatment for the acute respiratory distress syndrome: a multicentre, randomised controlled trial. Lancet Respir Med 8(3):267–276. 32043986 10.1016/S2213-2600(19)30417-5

[B237] Walls AC, Park YJ, Tortorici MA, Wall A, McGuire AT, Veesler D. 2020. Structure, Function, and Antigenicity of the SARS-CoV-2 Spike Glycoprotein. Cell 181(2):281–292 e6. 32155444 10.1016/j.cell.2020.02.058 PMC7102599

[B238] Wan S, Yi Q, Fan S, Lv J, Zhang X, Guo L, Lang C, Xiao Q, Xiao K, Yi Z, Qiang M, Xiang J, Zhang B, Chen Y, Gao C. 2020. Relationships among lymphocyte subsets, cytokines, and the pulmonary inflammation index in coronavirus (COVID-19) infected patients. Br J Haematol 189(3):428–437. 32297671 10.1111/bjh.16659 PMC7262036

[B239] Wang F NJ, Wang H, Zhao Q, Xiong Y, Deng L, Song S, Ma Z, Mo P, Zhang Y. 2020. Characteristics of peripheral lymphocyte subset alteration in COVID-19 pneumonia. J Infect Dis 221(11):1762–1769. 32227123 10.1093/infdis/jiaa150 PMC7184346

[B240] Wang H, Yang P, Liu K, Guo F, Zhang Y, Zhang G, Jiang C. 2008. SARS coronavirus entry into host cells through a novel clathrin- and caveolae-independent endocytic pathway. Cell Res 18(2):290–301. 18227861 10.1038/cr.2008.15 PMC7091891

[B241] Wang K, Chen W, Zhang Z, Deng Y, Lian J-Q, Du P, Wei D, Zhang Y, Sun X-X, Gong L, Yang X, He L, Zhang L, Yang Z, Geng J-J, Chen R, Zhang H, Wang B, Zhu Y-M, Nan G, Jiang J-L, Li L, Wu J, Lin P, Huang W, Xie L, Zheng Z-H, Zhang K, Miao J-L, Cui H-Y, Huang M, Zhang J, Fu L, Yang X-M, Zhao Z, Sun S, Gu H, Wang Z, Wang C-F, Lu Y, Liu Y-Y, Wang Q-Y, Bian H, Zhu P, Chen Z-N. 2020a. CD147-spike protein is a novel route for SARS-CoV-2 infection to host cells. Signal Transduct Target Ther 5(1):283. 33277466 10.1038/s41392-020-00426-x PMC7714896

[B242] Wang W, Su B, Pang L, Qiao L, Feng Y, Ouyang Y, Guo X, Shi H, Wei F, Su X, Yin J, Jin R, Chen D. 2020b. High-dimensional immune profiling by mass cytometry revealed immunosuppression and dysfunction of immunity in COVID-19 patients. Cell Mol Immunol 17(6):650–652. 32346099 10.1038/s41423-020-0447-2 PMC7186533

[B243] Wareing MD, Lyon AB, Lu B, Gerard C, Sarawar SR. 2004. Chemokine expression during the development and resolution of a pulmonary leukocyte response to influenza A virus infection in mice. J Leukoc Biol 76(4):886–895. 15240757 10.1189/jlb.1203644

[B244] Wilk AJ, Rustagi A, Zhao NQ, Roque J, Martínez-Colón GJ, McKechnie JL, Ivison GT, Ranganath T, Vergara R, Hollis T, Simpson LJ, Grant P, Subramanian A, Rogers AJ, Blish CA. 2020. A single-cell atlas of the peripheral immune response in patients with severe COVID-19. Nat Med 26(7):1070–1076. 32514174 10.1038/s41591-020-0944-y PMC7382903

[B245] Witsell AL, Schook LB. 1992. Tumor necrosis factor alpha is an autocrine growth regulator during macrophage differentiation. Proc Natl Acad Sci U S A 89(10):4754–4758. 1374912 10.1073/pnas.89.10.4754 PMC49162

[B246] Wolpe SD, Davatelis G, Sherry B, Beutler B, Hesse DG, Nguyen HT, Moldawer LL, Nathan CF, Lowry SF, Cerami A. 1988. Macrophages secrete a novel heparin-binding protein with inflammatory and neutrophil chemokinetic properties. J Exp Med 167(2):570–581. 3279154 10.1084/jem.167.2.570 PMC2188834

[B247] Wu C, Chen X, Cai Y, Xia J, Zhou X, Xu S, Huang H, Zhang L, Zhou X, Du C, Zhang Y, Song J, Wang S, Chao Y, Yang Z, Xu J, Zhou X, Chen D, Xiong W, Xu L, Zhou F, Jiang J, Bai C, Zheng J, Song Y. 2020a. Risk Factors Associated With Acute Respiratory Distress Syndrome and Death in Patients With Coronavirus Disease 2019 Pneumonia in Wuhan, China. JAMA Intern Med 180(7):934–943. 32167524 10.1001/jamainternmed.2020.0994 PMC7070509

[B248] Wu F, Zhao S, Yu B, Chen Y-M, Wang W, Song Z-G, Hu Y, Tao Z-W, Tian J-H, Pei Y-Y, Yuan M-L, Zhang Y-L, Dai F-H, Liu Y, Wang Q-M, Zheng J-J, Xu L, Holmes EC, Zhang Y-Z. 2020b. A new coronavirus associated with human respiratory disease in China. Nature 579(7798):265–269. 32015508 10.1038/s41586-020-2008-3 PMC7094943

[B249] Xie Y, Yu Y, Zhao L, Ning P, Luo Q, Zhang Y, Yin L, Zheng Y, Gao Z. 2021. Specific cytokine profiles predict the severity of influenza a pneumonia: a prospectively multicenter pilot study. Biomed Res Int 2021:9533044. 34692846 10.1155/2021/9533044 PMC8528594

[B250] Xu Z SL, Wang Y, Zhang J, Huang L, Zhang C, Liu S, Zhao P, Liu H, Zhu L, Tai Y, Bai C, Gao T, Song J, Xia P, Dong J, Zhao J, Wang FS. 2020. Pathological findings of COVID-19 associated with acute respiratory distress syndrome. Lancet 8(4):420–422. 10.1016/S2213-2600(20)30076-X PMC7164771 32085846

[B251] Yang Y, Shen C, Li J, Yuan J, Wei J, Huang F, Wang F, Li G, Li Y, Xing L, Peng L, Yang M, Cao M, Zheng H, Wu W, Zou R, Li D, Xu Z, Wang H, Zhang M, Zhang Z, Gao GF, Jiang C, Liu L, Liu Y. 2020. Plasma IP-10 and MCP-3 levels are highly associated with disease severity and predict the progression of COVID-19. J Allergy Clin Immunol 146(1):119–127.e4. 32360286 10.1016/j.jaci.2020.04.027 PMC7189843

[B252] Yokota S, Imagawa T, Mori M, Miyamae T, Aihara Y, Takei S, Iwata N, Umebayashi H, Murata T, Miyoshi M, Tomiita M, Nishimoto N, Kishimoto T. 2008. Efficacy and safety of tocilizumab in patients with systemic-onset juvenile idiopathic arthritis: a randomised, double-blind, placebo-controlled, withdrawal phase III trial. Lancet 371(9617):998–1006. 18358927 10.1016/S0140-6736(08)60454-7

[B253] Yoneyama M, Onomoto K, Jogi M, Akaboshi T, Fujita T. 2015. Viral RNA detection by RIG-I-like receptors. Curr Opin Immunol 32:48–53. 25594890 10.1016/j.coi.2014.12.012

[B254] Yu M, Qi W, Huang Z, Zhang K, Ye J, Liu R, Wang H, Ma Y, Liao M, Ning Z. 2015. Expression profile and histological distribution of IFITM1 and IFITM3 during H9N2 avian influenza virus infection in BALB/c mice. Med Microbiol Immunol 204(4):505–514. 25265877 10.1007/s00430-014-0361-2 PMC7087031

[B255] Yuen KY, Wong SS. 2005. Human infection by avian influenza A H5N1. Hong Kong Med J 11(3):189–199. 15951584

[B256] Zang R, Gomez Castro MF, McCune BT, Zeng Q, Rothlauf PW, Sonnek NM, Liu Z, Brulois KF, Wang X, Greenberg HB, Diamond MS, Ciorba MA, Whelan SPJ, Ding S. 2020. TMPRSS2 and TMPRSS4 promote SARS-CoV-2 infection of human small intestinal enterocytes. Sci Immunol 5(47):eabc3582. 32404436 10.1126/sciimmunol.abc3582 PMC7285829

[B257] Zanin M, Baviskar P, Webster R, Webby R. 2016. The interaction between respiratory pathogens and mucus. Cell Host Microbe 19(2):159–168. 26867175 10.1016/j.chom.2016.01.001 PMC4752725

[B258] Zhang J-M, An J. 2007. Cytokines, inflammation, and pain. Int Anesthesiol Clin 45(2):27–37. 17426506 10.1097/AIA.0b013e318034194e PMC2785020

[B259] Zhang Q, Raoof M, Chen Y, Sumi Y, Sursal T, Junger W, Brohi K, Itagaki K, Hauser CJ. 2010. Circulating mitochondrial DAMPs cause inflammatory responses to injury. Nature 464(7285):104–107. 20203610 10.1038/nature08780 PMC2843437

[B260] Zhang T, Kawakami K, Qureshi MH, Okamura H, Kurimoto M, Saito A. 1997. Interleukin-12 (IL-12) and IL-18 synergistically induce the fungicidal activity of murine peritoneal exudate cells against Cryptococcus neoformans through production of gamma interferon by natural killer cells. Infect Immun 65(9):3594–3599. 9284124 10.1128/iai.65.9.3594-3599.1997 PMC175511

[B261] Zhang Y-H, Zhao Y, Li N, Peng Y-C, Giannoulatou E, Jin R-H, Yan H-P, Wu H, Liu J-H, Liu N, Wang D-Y, Shu Y-L, Ho L-P, Kellam P, McMichael A, Dong T. 2013. Interferon-induced transmembrane protein-3 genetic variant rs12252-C is associated with severe influenza in Chinese individuals. Nat Commun 4(1):1418. 23361009 10.1038/ncomms2433 PMC3562464

[B262] Zhang YJ, Rutledge BJ, Rollins BJ. 1994. Structure/activity analysis of human monocyte chemoattractant protein-1 (MCP-1) by mutagenesis. Identification of a mutated protein that inhibits MCP-1-mediated monocyte chemotaxis. J Biol Chem 269(22):15918–15924. 8195247

[B263] Zheng M, Gao Y, Wang G, Song G, Liu S, Sun D, Xu Y, Tian Z. 2020. Functional exhaustion of antiviral lymphocytes in COVID-19 patients. Cell Mol Immunol 17(5):533–535. 32203188 10.1038/s41423-020-0402-2 PMC7091858

[B264] Zheng M, Karki R, Williams EP, Yang D, Fitzpatrick E, Vogel P, Jonsson CB, Kanneganti T-D. 2021. TLR2 senses the SARS-CoV-2 envelope protein to produce inflammatory cytokines. Nat Immunol 22:829–838. 33963333 10.1038/s41590-021-00937-x PMC8882317

[B265] Zhou P, Yang X-L, Wang X-G, Hu B, Zhang L, Zhang W, Si H-R, Zhu Y, Li B, Huang C-L, Chen H-D, Chen J, Luo Y, Guo H, Jiang R-D, Liu M-Q, Chen Y, Shen X-R, Wang X, Zheng X-S, Zhao K, Chen Q-J, Deng F, Liu L-L, Yan B, Zhan F-X, Wang Y-Y, Xiao G-F, Shi Z-L. 2020a. A pneumonia outbreak associated with a new coronavirus of probable bat origin. Nature 579(7798):270–273. 32015507 10.1038/s41586-020-2012-7 PMC7095418

[B266] Zhou Y, Fu X, Liu X, Huang C, Tian G, Ding C, Wu J, Lan L, Yang S. 2020b. Use of corticosteroids in influenza-associated acute respiratory distress syndrome and severe pneumonia: a systemic review and meta-analysis. Sci Rep 10(1):3044. 32080223 10.1038/s41598-020-59732-7 PMC7033254

[B267] Zhu N, Zhang D, Wang W, Li X, Yang B, Song J, Zhao X, Huang B, Shi W, Lu R, Niu P, Zhan F, Ma X, Wang D, Xu W, Wu G, Gao GF, Tan W, China Novel Coronavirus I, Research T. 2020a. A novel coronavirus from patients with pneumonia in China, 2019. N Engl J Med 382(8):727–733. 31978945 10.1056/NEJMoa2001017 PMC7092803

[B268] Zhu Z, Cai T, Fan L, Lou K, Hua X, Huang Z, Gao G. 2020b. Clinical value of immune-inflammatory parameters to assess the severity of coronavirus disease 2019. Int J Infect Dis 95:332–339. 32334118 10.1016/j.ijid.2020.04.041 PMC7195003

